# Molecular insights into TT2-type MYB regulators illuminate the complexity of floral flavonoids biosynthesis in *Freesia hybrida*

**DOI:** 10.1093/hr/uhae352

**Published:** 2024-12-12

**Authors:** Xiaotong Shan, Deyu Zhuang, Ruifang Gao, Meng Qiu, Liudi Zhou, Jia Zhang, Yanan Wang, Qi Zhang, Niu Zhai, Guoyun Xu, Li Wang, Yueqing Li, Xiang Gao

**Affiliations:** Key Laboratory of Molecular Epigenetics of MOE and Institute of Genetics & Cytology, Northeast Normal University, 5268 Renmin Street, Changchun 130024, Jilin, China; Key Laboratory of Molecular Epigenetics of MOE and Institute of Genetics & Cytology, Northeast Normal University, 5268 Renmin Street, Changchun 130024, Jilin, China; Key Laboratory of Molecular Epigenetics of MOE and Institute of Genetics & Cytology, Northeast Normal University, 5268 Renmin Street, Changchun 130024, Jilin, China; College of Plant Science, Jilin University, 5333 Xi'an Road, Changchun 130062, Jilin, China; Key Laboratory of Molecular Epigenetics of MOE and Institute of Genetics & Cytology, Northeast Normal University, 5268 Renmin Street, Changchun 130024, Jilin, China; Key Laboratory of Molecular Epigenetics of MOE and Institute of Genetics & Cytology, Northeast Normal University, 5268 Renmin Street, Changchun 130024, Jilin, China; Key Laboratory of Molecular Epigenetics of MOE and Institute of Genetics & Cytology, Northeast Normal University, 5268 Renmin Street, Changchun 130024, Jilin, China; Key Laboratory of Molecular Epigenetics of MOE and Institute of Genetics & Cytology, Northeast Normal University, 5268 Renmin Street, Changchun 130024, Jilin, China; Key Laboratory of Molecular Epigenetics of MOE and Institute of Genetics & Cytology, Northeast Normal University, 5268 Renmin Street, Changchun 130024, Jilin, China; China Tobacco Gene Research Center, Zhengzhou Tobacco Research Institute of CNTC, 2 Fengyang Street, Zhengzhou 450001, Henan, China; China Tobacco Gene Research Center, Zhengzhou Tobacco Research Institute of CNTC, 2 Fengyang Street, Zhengzhou 450001, Henan, China; Key Laboratory of Molecular Epigenetics of MOE and Institute of Genetics & Cytology, Northeast Normal University, 5268 Renmin Street, Changchun 130024, Jilin, China; Key Laboratory of Molecular Epigenetics of MOE and Institute of Genetics & Cytology, Northeast Normal University, 5268 Renmin Street, Changchun 130024, Jilin, China; Key Laboratory of Molecular Epigenetics of MOE and Institute of Genetics & Cytology, Northeast Normal University, 5268 Renmin Street, Changchun 130024, Jilin, China

## Abstract

Proanthocyanidins (PAs), anthocyanins, and flavonols are key flavonoids that play diverse roles in plant physiology and human health. Despite originating from a shared biosynthetic pathway, the regulatory mechanisms of PA biosynthesis and the cooperative regulation of different kinds of flavonoids remain elusive, particularly in flower tissues or organs. Here, we elucidated the regulatory network governing PA biosynthesis in *Freesia hybrida* ‘Red River®’ by characterizing four TT2-type MYB transcription factors, designated FhMYBPAs. Phylogenetic analysis, subcellular localization, and transactivation assays predicted their roles as PA-related activators. Pearson correlation analysis revealed significant correlations between FhMYBPAs and PA accumulation in various floral tissues and development stages. Functional studies demonstrated that FhMYBPAs activated PA biosynthesis by directly binding to the promoters of target genes, which can be enhanced by FhTT8L. Additionally, a hierarchical and feedback regulatory model involving FhTTG1, FhMYB27, and FhMYBx was proposed for PA biosynthesis. Furthermore, comparative analysis of flavonoid-related MYB factors involving FhPAP1, FhMYB5, FhMYBF1, and FhMYB21L2 highlighted their roles in regulating PA, anthocyanin, and flavonol biosynthesis, with some exhibiting versatile regulations. Overall, our findings provide insights into the spatio-temporal regulation of flavonoids in flowers and expand our understanding of MYB-mediated transcriptional regulation of specialized metabolites in plants.

## Introduction

Proanthocyanidins (PAs), or condensed tannins, are a class of flavonoids formed through the polymerization of flavan-3-ols. Widely distributed across plant tissues like bark, seeds, fruits, and leaves, PAs play crucial roles in plant defense against various stresses like UV radiation, cold, pathogens, and herbivores [1–3]. PAs also contribute astringency and bitterness to foods and beverages like wine, tea, and berries, affecting flavor profiles and consumer appeal [4]. Additionally, PAs serve as antioxidants, benefiting cardiovascular and neurological health, and hold promise for applications in nutraceuticals, livestock, and food industries [5–7]. Consequently, a comprehensive understanding of PA biosynthesis and regulation in plants is essential.

PA biosynthesis is a specialized branch of the flavonoid pathway, sharing precursors with anthocyanins and flavonols. Various flavan-3-ols, catalyzed by PA-specific enzymes such as leucoanthocyanidin reductase (LAR) and anthocyanidin reductase (ANR), are the building blocks for PAs. PA biosynthesis is tightly regulated at the transcriptional level in a tissue- and developmental stage-specific manner, involving transcription factors from at least seven families including MYB, bHLH, WD40, WRKY, MADS-box, zinc fingers, and HD-ZIP [8–12]. The MYB-bHLH-WD40 (MBW) complex plays a pivotal role in controlling PA production by activating key genes like *DFR*, *ANS*, *LAR*, and *ANR* (*BAN*) [13–15], where bHLH and WD40 also participate in various biological pathways [16]. Similar PA-related MBW complexes have been identified in many species [3, 11]. Notably, the PA biosynthesis-related MYB activators can be broadly categorized into three types: TT2 type (represented by *Arabidopsis thaliana* AtTT2), MYB5 type (represented by AtMYB5), and PA1 type (represented by *Vitis vinifera* VvMYBPA1), each exhibiting distinct sequence and functional characteristics [17, 18].

The components of the MBW complex involved in PA biosynthesis usually exhibit mutual transcriptional regulation, although this phenomenon has been reported in only a limited number of plant species. For instance, in *Medicago truncatula*, MtWD40–1 is activated by PA-related MtMYB5 and MtMYB14 in the presence of MtTT8 and MtWD40 [15]. The PA regulatory network is further complicated by the discovery of MYB repressors, which can downregulate PA biosynthesis by competing with MYB activators for bHLH binding [9, 19–21]. Consequently, possible hierarchical and feedback regulation may fine-tune PA biosynthesis. However, the detail mechanisms require further clarification. Moreover, the research on PA biosynthesis regulation has mainly focused on vegetative organs, seeds, and fruits of PA-rich model or crop plants [3, 11]; knowledge of regulatory conservation in flowers remains limited especially in monocot plants.

Flowers, crucial for ornamental traits and reproductive success, also benefit from PAs. These compounds protect flowers from UV radiation due to strong UV absorption [22] and may produce red anthocyanins under certain conditions, adjusting flower coloration. Furthermore, the presence of flavonols, another kind of flavonoids, in flowers enhances pollen viability, germination, tube growth, and pollinator attraction [23]. Given that PAs, anthocyanins, and flavonols share a common biosynthetic pathway [18, 24–26], how the transcription factors synchronize the production of these different flavonoids within an organ remain unclear.

*Freesia hybrida*, a monocot in the Iridaceae family, is popular for its elegant, fragrant blooms [27, 28]. Studies have shown that flowers from various cultivars accumulate high levels of flavonoids, including anthocyanins, PAs, and flavonols [29–32]. In *F. hybrida* ‘Red River®’, the hierarchical and feedback regulatory networks governing anthocyanin biosynthesis have been elucidated, involving the R2R3-MYB activator FhPAP1, bHLH factors FhTT8L and FhGL3L, and the WD40 protein FhTTG1 [29, 31, 33], as well as MYB repressors FhMYB27 and FhMYBx [34]. Comparatively, flavonol biosynthesis is primarily regulated by FhMYBF1/2/3/4 from MYB subgroup 7 (SG7) and FhMYB21 from SG19 [32]. Moreover, FhMYB5, an ortholog of AtMYB5 [35], plays a role in the general flavonoid pathway [30]. However, the specific R2R3-MYB activators involved in PA biosynthesis, along with their cooperation with above MYB factors in regulating anthocyanins, flavonols, and PAs, remain poorly understood in this plant.

In this study, four TT2-type R2R3-MYB transcription factors, FhMYBPAs, from *F. hybrida* were functionally characterized and their roles in the hierarchical and feedback regulatory network governing PA biosynthesis were also elucidated. Moreover, the synergistic regulatory roles of all characterized *Freesia* flavonoid-related MYB proteins were systematically compared. This comprehensive investigation aims to enhance our understanding of PA regulation in plants and provides new insights into the synergistic regulation of different flavonoids in flowers. Additionally, functional MYB genes identified here could be utilized to modify PA content for ornamental or therapeutic applications.

## Results

### *FhMYBPAs* encode TT2-type MYB transcription factors

**Figure 1:**
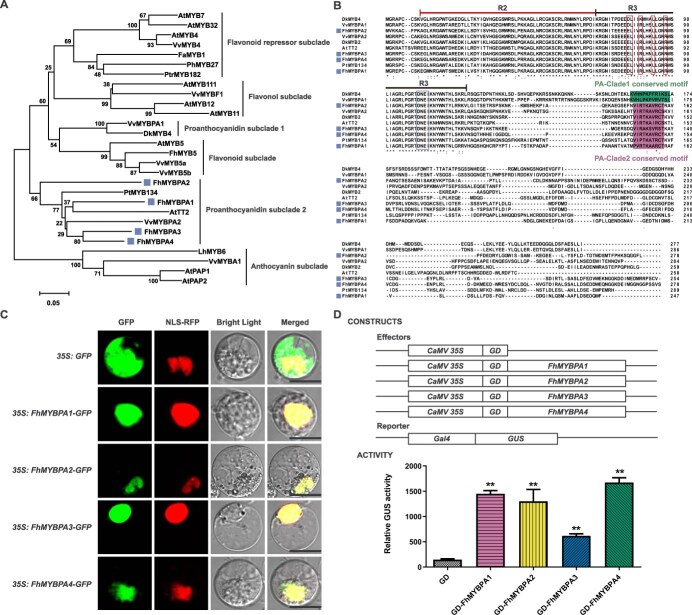
FhMYBPAs are TT2-type R2R3 MYB transactivators. A. Phylogenetic analysis of FhMYBPAs and other flavonoid related MYB regulators. The tree was constructed using neighbor-joining algorithm in MEGA X. Bootstrapping with 1000 replications was performed. Sequences from *Freesia* were indicated by squares. The Genbank accession numbers were as follows: *A. thaliana* AtMYB7 (NP_179263), AtMYB32 (NP_195225), AtMYB4 (AAC83582), AtMYB11 (NP_191820), AtMYB12 (CAB09172), AtMYB111 (NP_199744), AtMYB5 (U26935), AtTT2 (Q9FJA2), AtPAP1 (AAG42001), AtPAP2 (NP_176813); *V. vinifera* VvMYB4 (NP_001268129), VvMYBF1 (FJ948477), VvMYB5a (AAS68190), VvMYB5b (Q58QD0), VvMYBPA1 (AM259485), VvMYBPA2 (ACK56131), VvMYBA1 (BAD18977); *Fragaria ananassa* FaMYB1 (AAK84064); *Petunia hybrida* PhMYB27 (AHX24372); *Populus tremula × Populus tremuloides* PtrMYB182 (AJI76863), PtrMYB134 (ACR83705); *Diospyros kaki* DkMYB4 (BAI49721); *F. hybrida* FhMYB5 (QAX87835); *Lilium hybrid* LhMYB6 (BAJ05399). B. Multiple alignment of the amino acid sequences of FhMYBPAs and PA-related MYB activators in other species. The R2 and R3 domains were indicated by overlines, respectively. The conserved motif [D/E]Lx2[R/K]x3Lx6Lx3R responsible for interaction with bHLH factors and the ‘DNEI’ consensus motif, characteristic of PA-related MYB factors, were outlined by different rectangular frames. The shaded residues in the C-termini of various MYBs indicated the conserved motif in different PA clade factors, respectively. Numbers indicated the position of the last amino acid in each line. *, Identical amino acids; : or . , similar amino acids. Squares indicated the *Freesia* PA-related MYBs isolated in this study. C. Subcellular localization of FhMYBPAs. Constructs indicated on the left were cotransfected with nuclear localization signal (NLS)-fused RFP into *Freesia* protoplasts. From left to right panels in sequence: GFP channel, RFP channel, bright field image, merged image. GFP and RFP fluorescence was detected under a laser-scanning confocal microscope. At least 10 cells were observed, and similar results were obtained. Scale bar = 25 μm. D. Transactivation capacities of FhMYBPAs. Effectors and reporters for transient assays were shown by schematic diagrams. Data represented the mean ± SD of three replicates. Student’s *t* test was used to analyze the significant difference (^**^P < 0.01).

Utilizing AtTT2 (TT2 type) and VvMYBPA1 (PA1 type) as query sequences in a BLAST search against the *Freesia* transcriptome database, we identified four putative PA-related MYB genes, designated as *FhMYBPA1*, *FhMYBPA2*, *FhMYBPA3*, and *FhMYBPA4* (Table S1). Phylogenetic analysis revealed that the FhMYBPAs clustered within the TT2-type MYB subclade, establishing their classification as TT2-type MYB proteins (Fig. 1a). Amino acid sequence alignments showed that the R2R3 repeat regions of FhMYBPAs exhibited high homology with other MYB factors and contained the conserved [D/E]Lx2[R/K]x3Lx6Lx3R motif, essential for bHLH protein interaction [36, 37]. Additionally, the adjacent ‘DNEI’ consensus motif, characteristic of PA-related MYB factors, was consistently identified (Fig. 1b). All FhMYBPAs contained the VI[R/P]TKAx1RC[S/T] motif, conserved in TT2-type MYBs but absent in PA1-type factors (Fig. 1b). Interestingly, MEME analysis (https://meme-suite.org/meme/tools/meme) revealed that a G-28 motif, typically found in PA1-type MYBs, also present in FhMYBPA2 (Fig. S1).

The subcellular localizations of FhMYBPAs were investigated in *Freesia* protoplasts. The resulting FhMYBPAs-GFP fusion proteins localized exclusively to the nucleus, indicating their role as transcriptional regulators (Fig. 1c). Additionally, Gal4-based transient transfection assays verified the transactivation properties of all four FhMYBPAs (Fig. 1d). These findings indicated we had cloned four PA-related MYB activators, facilitating an in-depth exploration of the regulatory mechanisms underlying floral PA biosynthesis in *F. hybrida*.

### FhMYBPAs positively correlate with *Freesia* PA biosynthesis

**Figure 2:**
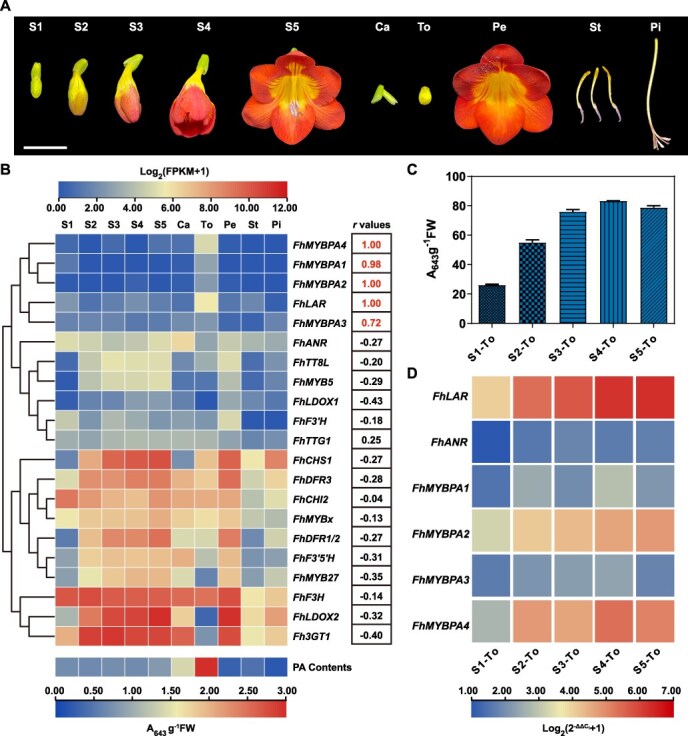
FhMYBPAs are positively correlated with PA accumulation *F. hybrida* ‘Red River®’. A. *Freesia* flowers at five developmental stages and different tissues stripped from fully blooming flowers. S1 (Stage 1), <10 mm long with unpigmented buds; Stage 2, 10–20 mm long with slightly pigmented buds. Stage 3, 20–30 mm long with pigmented buds. Stage 4, fully pigmented flowers before complete opening. Stage 5, fully opened flowers. Ca, calyx; Pe, petal; Pi, pistil. St, stamen; To, torus (The torus is enclosed by the calyx in *Freesia* flowers, and the petals, pistil, and stamens are attached to the torus.). Bars, 2 cm. B. Patterns of flavonoid-related gene expression and PA accumulation at different flower developmental stages and tissues in *F. hybrida* ‘Red River®’ (left panel) and Pearson correlation analysis between the genes and PA (right panel). FPKM values from RNA-seq were normalized by log_2_(FPKM+1) and hierarchically clustered using the average Pearson distance metric. PA contents were quantified as A_643_ g^−1^ fresh weight (FW), and the data represent the mean value of three independent experiments. C. PA contents of torus at different flower developmental stages. D. Expression patterns of *FhMYBPAs* and PA-specific genes *FhLAR* and *FhANR* in torus at different flower developmental stages. The mean data from RT-qPCR of at least three biological replicates were normalized as log_2_(2^-ΔΔC^_t_ + 1).

To clarify the involvement of FhMYBPAs in *Freesia* PA biosynthesis, we analyzed the relative expression levels of flavonoid-related genes across different flower developmental stages (Stage 1–5, S1–S5) and floral tissues (from S5) of *F. hybrida* ‘Red River®’ (Fig. 2a). Notably, these genes separated into at least two distinct clades based on expression patterns. Anthocyanin biosynthesis-related genes, including *FhCHS1*, *FhCHI2*, *FhF3H*, *FhF3’5’H*, *FhDFRs*, *FhLDOX2*, and *Fh3GT1*, formed a cohesive group (Fig. 2B). In contrast, the four *FhMYBPAs* clustered with PA-related genes such as *FhLAR* and *FhANR*, as well as previously characterized *FhMYB5* and *FhTT8L* [29, 30], suggesting that FhMYBPAs play a regulatory role in PA biosynthesis.

Furthermore, the anthocyanin-related gene cluster showed relatively higher expression levels in most examined samples compared to the putative PA pathway-related genes, consistent with the prominent pigmentation observed in *Freesia* flowers (Fig. 2b). Pearson coefficients calculated between these genes and PA content revealed significant correlations for the four *FhMYBPAs* and *FhLAR* with PA accumulation, highlighting their roles as key activators in *Freesia* PA biosynthesis (Fig. 2b). Interestingly, PA content analysis in various tissues indicated that torus accumulated the highest PA levels (Fig. 2b). PA content in the torus increased with flower development, suggesting a critical, previously uncharacterized role of PAs in this tissue (Fig. 2c). *FhLAR*, *FhMYBPA2*, and *FhMYBPA4* exhibited relatively higher and progressively increasing expression in the developing torus, underscoring their essential roles in PA accumulation within this floral structure (Fig. 2d).

### FhMYBPAs positively regulate PA biosynthesis in plants

**Figure 3:**
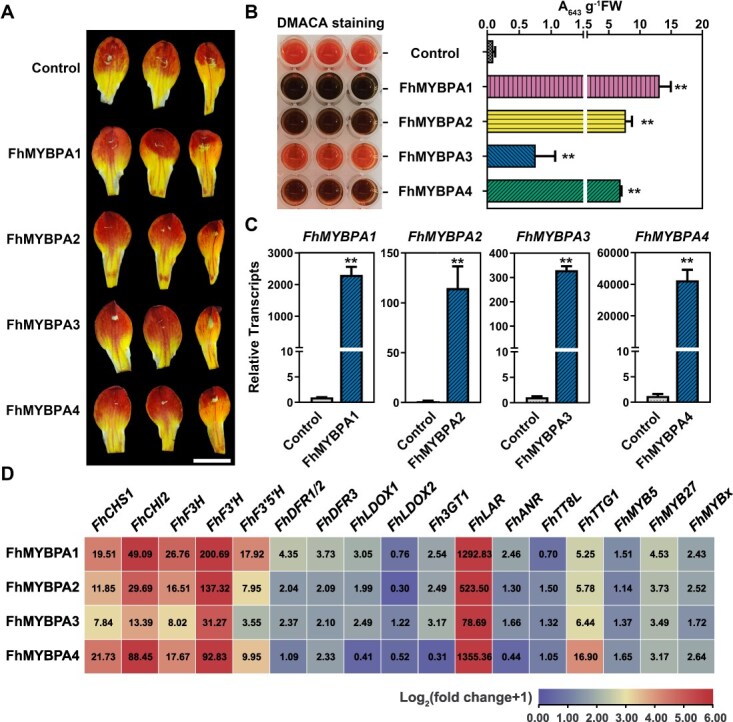
Transient overexpression of FhMYBPAs in petals of *F. hybrida* ‘Red River®’ promotes PA accumulation. **A.** Phenotypes of *Freesia* petals transiently overexpressing FhMYBPAs. B. DMACA staining and quantification of PA in FhMYBPA-transfected *Freesia* petals. Data represent mean ± SD of three biological replicates. C. Relative expression levels of *FhMYBPAs* in transfected *Freesia* petals detected by RT-qPCR. Data represent mean ± SD of three biological replicates. D. Fold changes of flavonoid-related genes in FhMYBPA-transfected *Freesia* petals. The mean data from RT-qPCR of at least three biological replicates were normalized as 2^-ΔΔC^_t_.

To investigate the functions of FhMYBPAs *in vivo*, we transiently expressed these genes in the petals of *F. hybrida* ‘Red River®’ using *Agrobacterium* infiltration (Fig. 3a). Dimethylaminocinnamaldehyde (DMACA) staining visualized PA accumulation, revealing a significant increase in PAs within petals overexpressing *FhMYBPAs* (Figs. 3b and 3c). Reverse transcription quantitative polymerase chain reaction (RT-qPCR) analysis revealed that most biosynthetic genes were upregulated, with the PA-specific *FhLAR* gene showing the most pronounced increase level (Fig. 3d). Interestingly, flavonoid regulators such as FhTTG1, FhMYB27, and FhMYBx were also markedly upregulated (Fig. 3d). These results suggested that all four FhMYBPAs function as R2R3-MYB activators in *Freesia* PA biosynthesis. Among them, FhMYBPA2 appears particularly effective, as it significantly enhanced PA accumulation despite its relatively lower expression levels in transient assays. This finding implies that FhMYBPA2 might be a prime candidate for activating PA biosynthesis.

To further characterize FhMYBPAs, we performed stable overexpression in *Nicotiana tabacum* ‘K326’. While no observable phenotypic changes were noted in transgenic tobacco flowers (Figs. S2a and S2b), flowers overexpressing *FhMYBPAs* displayed higher PA levels than controls (Fig. S2c). DMACA staining confirmed PA presence in various floral tissues, including calyx, petals, and stamens (Fig. S2d). Further RT-qPCR analysis showed notable upregulation of key PA biosynthetic genes (*NtDFR*, *NtLAR*, and *NtANR)* in transgenic flower tissues (Fig. S2e). These findings suggested that FhMYBPAs also enhance PA biosynthesis in tobacco by activating PA pathway genes.

### FhMYBPAs interact with FhTT8L to activate proanthocyanidin biosynthetic genes in *Freesia*

**Figure 4:**
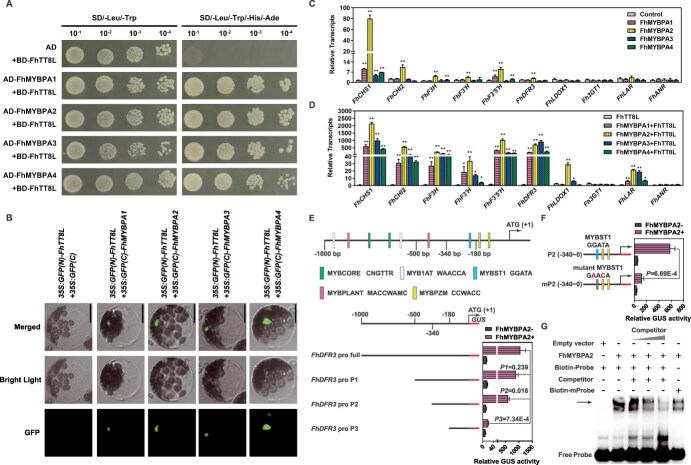
FhMYBPAs promote PA biosynthesis by directly binding to target gene promoters, a process enhanced by FhTT8L. **A.** Y2H analysis of the interaction between FhMYBPAs and FhTT8L. The AH109 yeast strain was cotransformed with the indicated combinations of FhMYBPAs and FhTT8L fused to AD and BD, respectively. Transformants were plated on synthetic dropout (SD) medium without leucine or tryptophan. Further validation of the interaction was carried out on SD medium without leucine, tryptophan, histidine, or adenine. The values 10^−1^, 10^−2^, 10^−3^, and 10^−4^ indicate yeast dilution concentrations of 10, 100, 1000, and 10 000 times, respectively. B. BiFC assay of the interaction between FhMYBPAs and FhTT8L. *GFP(N)-FhTT8L* and *GFP(C)-FhMYBPAs* or *GFP(N)-FhTT8L* and *GFP(N)* constructs were cotransformed into *Arabidopsis* protoplasts to detect the interaction between FhMYBPAs and FhTT8L *in planta*. GFP fluorescence was detected under a laser-scanning confocal microscope. At least 10 cells were observed. Scale bar = 25 μm. C and D. Relative transcripts of flavonoid biosynthetic genes in *Freesia* protoplasts overexpressing FhMYBPAs alone (C) or in combination with FhTT8L (D). Data represent the mean ± SD of three replicates. Asterisks (^*^P < 0.05; ^**^P < 0.01) indicate values determined by Student’s *t*-test. E. Schematic representation and truncation analysis of *FhDFR3* promoter. The rectangular boxes with different colors indicate various MYB-binding sites. The numbers displayed in the schematic indicate the distance from the *FhDFR3* translation initiation site. Data represent means±SD (*n* = 3, transfection experiments were performed three times). *P*-values were calculated versus the GUS activity driven by the full-length promoter activated by FhMYBPA2 (Student’s *t*-test). F. Promoter activation effects of FhMYBPA2 on wild-type and mutant MYBST1 binding site in *FhDFR3* promoter. Left, schematic diagram of mutant MYBST1 in truncated *FhDFR3* promoter (−340–0 bp). Right, promoter activities measured in the presence or absence of FhMYBPA2. Data represent means±SD (*n* = 3, transfection experiments were performed three times). Statistical analysis was performed by Student’s *t*-test. G. EMSA analysis showing that FhMYBPA2 bound to the MYBST1 site in the *FhDFR3* promoter *in vitro*. The black arrow denotes the shifted probe.

It is widely known that TT2-type MYB activators typically interact with TT8-like bHLH factors to regulate PA biosynthesis. Alignment analysis of FhMYBPAs revealed a conserved bHLH-binding domain in their R3 repeats (Fig. 1b), prompting further exploration of potential interactions with FhTT8L. In yeast two-hybrid (Y2H) assays, only yeast cells coexpressing FhMYBPAs with an activation domain (AD) and FhTT8L with a binding domain (BD) grew on selective media (SD/−Trp-Leu-His-Ade) (Fig. 4a). This interaction was further validated by bimolecular fluorescence complementation (BiFC) assays (Fig. 4b). Additional evidence was obtained through the Gal4-based system, where significant β-glucuronidase (GUS) activity was observed only when HA-FhMYBPAs were coexpressed with GD-FhTT8L (Fig. S3a), but not with the WD40 protein FhTTG1 (Fig. S3b).

To investigate the effects of FhTT8L on FhMYBPA-mediated PA biosynthesis, FhMYBPAs were transfected alone or in combination with FhTT8L into *Freesia* protoplasts. Cotransfection of FhMYBPAs with FhTT8L significantly enhanced the activation of general flavonoid pathway genes and *FhLAR* compared to FhMYBPAs alone (Figs. 4c and 4d). However, neither FhMYBPAs alone nor in combination with FhTT8L activated the anthocyanin-related *Fh3GT1* or PA-related *FhANR* (Figs. 4c and 4d). To further examine this mechanism, FhMYBPA2 was selected for transfection alone or with FhTT8L into *Arabidopsis* protoplast to evaluate the activation on promoters of *Freesia* PA biosynthetic genes, and the results obtained were consistent with the above findings (Fig. S4).

To determine if FhMYBPAs directly bind to target promoters, we analyzed the *FhDFR3* promoter for potential MYB-binding sites using the New PLACE tool (https://www.dna.affrc.go.jp/PLACE/?action=newplace) (Fig. 4e). Further transient protoplast assays revealed a notable drop in GUS activity upon truncation from *FhDFR3*proP2 to *FhDFR3*proP3, indicating that the MYBST1 site in this region is crucial for recognition by FhMYBPA2 (Fig. 4e). The pivotal role of MYBST1 was further validated by sequence mutation, which resulted in an obvious decrease in GUS activation by MYBPA2 (Fig. 4f). Subsequently, the direct binding of FhMYBPA2 to the *FhDFR3* promoter was validated by electrophoretic mobility shift assay (EMSA) (Fig. 4g and Fig. S5). Collectively, these results indicate that FhMYBPAs can activate *Freesia* PA biosynthetic genes by directly binding to their promoters, with this process strengthened through interaction with FhTT8L.

### *Freesia* PA biosynthesis is regulated by a hierarchical and feedback loop

**Figure 5:**
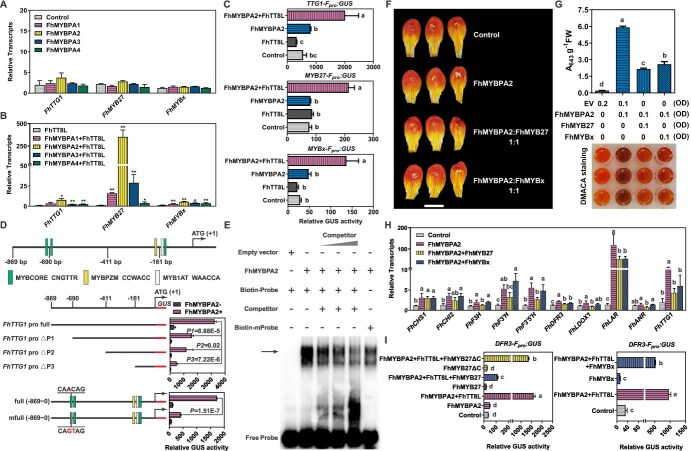
*Freesia* PA biosynthesis is regulated in a hierarchical and feedback loop. A and B. Relative transcripts of *FhTTG1*, *FhMYB27*, and *FhMYBx* in *Freesia* protoplasts overexpressing FhMYBPAs alone or in combination with FhTT8L. Data represent the mean ± SD of three replicates. Asterisks (^*^P < 0.05; ^**^P < 0.01) indicate values determined by Student’s *t*-test. C. Activation effects of FhMYBPA2 and FhTT8L on promoters of *FhTTG1*, *FhMYB27*, and *FhMYBx*. *GUS* was driven by the respective promoter as a reporter. FhMYBPA2 was transfected alone or cotransfected with FhTT8L as effectors. Rosette leaves from 4-week-old *Arabidopsis* plants were used for protoplast isolation. Each column represents the mean value of three replicates with error bars indicating SD. One-way analysis of variance (ANOVA) was carried out to compare statistical differences (Ducan, *P* < 0.05). D. Schematic representation and truncation or mutation analysis of the *FhTTG1* promoter. The rectangular boxes with different colors indicate various MYB-binding sites. The numbers displayed in the schematic indicate the distance from the *FhTTG1* translation initiation site. Data represent means±SD (*n* = 3, transfection experiments were performed three times). *P*-values were calculated versus the GUS activity driven by the full-length promoter activated by FhMYBPA2 (Student’s *t*-test). E. EMSA analysis showing that FhMYBPA2 bound to the MYBCORE site in the *FhTTG1* promoter *in vitro*. The black arrow denotes the shifted probe. F. Phenotypes of *Freesia* petals transiently overexpressing FhMYBPAs alone or in combination with FhMYB27 or FhMYBx. G. DMACA staining and quantification of PAs in *Freesia* petals overexpressing FhMYBPAs alone or in combination with FhMYB27 or FhMYBx. Data represent mean ± SD of three biological replicates. One-way ANOVA was carried out to compare statistical differences (Ducan, *P* < 0.05). H. Relative transcripts of flavonoid biosynthetic genes detected by RT-qPCR in *Freesia* petals overexpressing FhMYBPAs alone or in combination with FhMYB27 or FhMYBx. Data represent the mean ± SD of three replicates. One-way ANOVA was carried out to compare statistical differences (Ducan, *P* < 0.05). I. Transient expression assay showing that FhMYB27 and FhMYBx were corepressors of the MBW activation complex. The reporter construct *DFR3-F_pro_:GUS* contained a *GUS* reporter gene driven by *FhDFR3* promoter. *DFR3-F_pro_:GUS* was cotransfected into *Arabidopsis* protoplasts together with the effector constructs diagrammed under the pillars. FhMYB27ΔC is a truncated version of FhMYB27 with its C-terminal repression domain removed. Data represent the mean ± SD of three replicates. One-way ANOVA was carried out to compare statistical differences (Ducan, *P* < 0.05).

As mentioned above, transient overexpression of FhMYBPAs in *F. hybrida* ‘Red River®’ petals upregulated *FhTTG1*, *FhMYB27*, and *FhMYBx* by several folds (Fig. 3d), suggesting a potential hierarchical or feedback loop among these proteins. To further investigate their interactions, FhMYBPAs were transiently overexpressed in *Freesia* protoplasts alone or in combination with FhTT8L. Results showed that FhMYBPAs alone hardly activated *FhTTG1*, *FhMYB27*, or *FhMYBx* (Fig. 5a). However, cotransfection of FhMYBPAs with FhTT8L significantly enhanced activation of these genes in most cases (Fig. 5b). The activation of *FhTTG1*, *FhMYB27*, and *FhMYBx* by the FhMYBPAs-FhTT8L complex was further validated using a GUS reporter system driven by their promoters with different combinations of FhMYBPA2 and FhTT8L (Fig. 5c).

To pinpoint potential FhMYBPA binding sites, activation effects of FhMYBPA2 on variously truncated *FhTTG1* promoters were tested in the presence of FhTT8L. Truncation analysis indicated that MYB-binding sites around −690 bp upstream of the ATG start codon played crucial roles (Fig. 5d). Mutation of the MYBCORE motif within this region significantly reduced GUS activity (Fig. 5d), and EMSA further confirmed that the MYBCORE motif served as the direct binding site for FhMYBPA2 in the *FhTTG1* promoter (Fig. 5e).

To assess the role of FhMYB27 and FhMYBx in PA biosynthesis, coinfiltration experiments with *FhMYBPA2* were conducted in *F. hybrida* ‘Red River®’ petals (Fig. 5f). Results showed that FhMYB27 and FhMYBx significantly suppressed PA accumulation (Fig. 5g), suggesting their involvement in a feedback loop for PA regulation. RT-qPCR analysis revealed that most PA biosynthetic genes activated by FhMYBPA2 were notably repressed by FhMYB27 or FhMYBx (Fig. 5h). To clarify their repressive mechanisms, GUS reporter assays with *FhDFR3* and *FhTTG1* promoters, along with FhMYBPA2 and FhTT8L, were performed. Consistent with previous findings, both promoters were strongly activated by the FhMYBPA2-FhTT8L complex, but this activation was significantly repressed when FhMYB27 or FhMYBx was cotransfected (Fig. 5i and Fig. S6). Moreover, our earlier study revealed that the C-terminal repression domain conferred strong transrepression capacity to FhMYB27 [34]. A truncated version, FhMYB27ΔC [34], lacking this domain, showed substantially reduced repression of *FhDFR3* promoter activity activated by FhMYBPA2 and FhTT8L, highlighting the importance of the repression domain in FhMYB27 in regulating PA biosynthesis (Fig. 5i). Together, these findings demonstrate that *Freesia* PA biosynthesis is regulated by a hierarchical and feedback loop mechanism.

### Functional pleiotropy and differentiation of MYB factors in regulating different types of flavonoids

**Figure 6:**
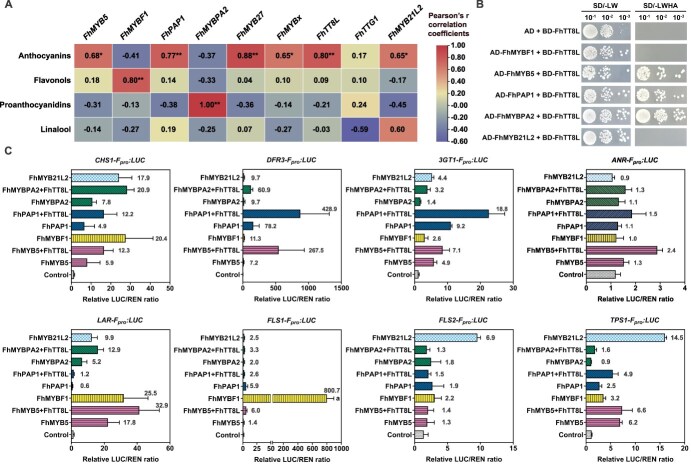
Differentiation of flavonoid-related MYBs from *F. hybrida* ‘Red River®’. A. Pearson correlation analysis between expression patterns of flavonoid-related factors and metabolite accumulations in flowers at different developmental stages and floral tissues of *F. hybrida* ‘Red River®’. Pearson’s *r* correlation coefficients comparing the transcript levels of each gene to specific metabolite content are shown in the heat map. *r-*values with significant *P*-values (^*^P ≤ 0.05, ^**^P ≤ 0.01) are highlighted. B. Y2H analysis of the interaction between different MYBs and FhTT8L. The AH109 yeast strain was cotransformed with the indicated combinations of MYBs and FhTT8L fused to AD and BD, respectively. Transformants were plated on SD medium without leucine or tryptophan. Further validation of the interaction was carried out on SD medium without leucine, tryptophan, histidine, or adenine. The values 10^−1^, 10^−2^, and 10^−3^ indicate yeast dilution concentrations of 10, 100, and 1000 times, respectively. C. Activation effects of FhMYBs on selected promoters detected in *Arabidopsis* protoplasts. Promoters of *FhCHS1* and *FhDFR3*, representatives of EBGs and LBGs of the flavonoid pathway, as well as promoters of *Fh3GT1*, *FhLAR*, and *FhANR*, and two *FhFLSs*, representatives of anthocyanin-, PA-, and flavonol-specific genes, were selected to drive *LUC* reporter*.* The 35S promoter driving *REN* was used as an internal control. *FhTTG1* was transfected as background when detecting *FhANR* promoter activities. Data represent means±SD (*n* = 3, transfection experiments were performed three times). The number to the right of each bar represents the fold change of this combination compared to the control.

To date, MYB factors from at least seven MYB subfamilies have been characterized in the regulation of anthocyanins, flavonols, or PAs in *F. hybrida* ‘Red River®’. To systematically compare their functional pleiotropy and differentiation, Pearson correlation analysis was performed between MYB expression patterns and metabolite accumulation across different flower development stages and tissues (Fig. 6a and Fig. S7). The results showed significant correlations of *FhPAP1*, *FhMYB27*, and *FhTT8L* with anthocyanin biosynthesis, while *FhMYBF1* and *FhMYBPA2* correlated specifically with flavonols and PAs, respectively. Comparatively, *FhMYB5*, *FhMYBx*, *FhMYB21L2*, as well as *FhTTG1* had relatively lower correlations, suggesting their more versatile or less dominant roles in regulating those metabolites (Fig. 6a).

To better define the collaborative or specific regulations of these MYBs on flavonoid pathway genes, the Y2H system was initially used to confirm the interactions between MYB proteins and FhTT8L (Fig. 6b). Dual-luciferase reporter assays were then conducted using promoters of *FhCHS1* and *FhDFR3* (representing early and late biosynthetic genes, EBGs and LBGs), along with promoters for anthocyanin (*Fh3GT1*), PA (*FhLAR*, *FhANR*), and flavonol (*FhFLS1*, *FhFLS2*) pathway-specific genes. Results indicated that nearly all MYBs activated *FhCHS1*, while FhPAP1 and FhMYB5 exhibited stronger activation on *FhDFR3* (Fig. 6c). Consistent with the pivotal roles of FhPAP1 in regulating anthocyanin biosynthesis and FhMYBF1 and FhMYB21L2 in flavonol biosynthesis, *Fh3GT1* was most significantly activated by FhPAP1, whereas *FhFLS1* and *FhFLS2* were mainly activated by FhMYBF1 and FhMYB21L2, respectively (Fig. 6c). In comparison, both FhMYB5 and FhMYBPA2 activated *FhLAR*, while the activation of *FhANR* required the MYB5-FhTT8L-FhTTG complex. Interestingly, FhMYBF1 and FhMYB21L2 also activated *FhLAR*, suggesting a potential role in balancing flavonol and PA biosynthesis (Fig. 6c). Overall, these findings indicate that while MYB factors have specific roles in regulating particular metabolites, some exhibit pleiotropic effects across different metabolite pathways.

## Discussion

### Functional diversity of TT2- and MYB5-type MYB activators in PA biosynthesis in *Freesia* flower

The regulation of PA biosynthesis by the MBW complex is conserved across various dicots, including model plants like *Arabidopsis*, as well as some horticultural species [3, 17, 26, 38]. Our findings confirm that this regulatory module is similarly conserved in *F. hybrida*, a monocot flowering plant. Within this complex, R2R3-MYB activators play a crucial role in controlling gene expression by binding directly to promoter DNA [39]. PA biosynthesis involves at least three types of MYB activators: TT2-type, MYB5-type, and PA1-type [18]. TT2-type MYBs are well characterized and critical for PA accumulation across different plant tissues [17, 40–43], while MYB5-type factors show more functional diversity. For instance, *Arabidopsis* MYB5 contributes primarily to seed mucilage synthesis and trichome formation, with a minor role in PA production [35, 44]. *Vitis vinifera* VvMYB5a regulates multiple metabolites including anthocyanins, flavonols, and PAs [45]. Comparatively, PA1-type MYBs have only been characterized in dicots [46–49]. In our study, we identified four TT2-type MYB transcription factors in *Freesia*, yet no PA1-type MYBs were found (Fig. 1 and Table S1). Interestingly, one TT2-type MYB, FhMYBPA2, contains a G-28 motif typically found in PA1-type MYBs [18], suggesting an evolutionary link between TT2-type and PA1-type MYB regulators (Fig. S1).

The PA biosynthetic pathway dates back to early terrestrial plants, and evolutionary processes like genome duplication and chromosomal rearrangement have led to gene duplication and functional divergence, yielding distinct multigene families [50]. Key genes like *DFR*, *LAR*, and *ANR* share a common origin but have diversified roles within the flavonoid biosynthetic pathway. In *Arabidopsis*, most PA-related genes exist as single copy, whereas angiosperms such as poplar and grapevine exhibit multiple gene copies with distinct regulatory mechanisms [10, 46, 51, 52]. Our findings reveal distinct roles among the PA-related MYB activators in *Freesia*. Notably, *FhMYBPA2* and *FhMYBPA4* exhibit increased expression during torus development, suggesting their involvement in PA accumulation, while FhMYB5 may regulate PA biosynthesis in other tissues (Fig. 2). Additionally, while both FhMYB5 and FhMYBPAs activate general flavonoid pathway genes, they demonstrate specificity in regulating *FhLAR* and *FhANR* expressions (Fig. 6).

### Hierarchical regulation and feedback mechanisms in flavonoid biosynthesis

MBW complexes intricately regulate plant growth, development, and metabolism through specific MYB transcription factors. In *Arabidopsis*, these MYB factors direct processes like anthocyanin accumulation, PA biosynthesis, and trichome development, which is enhanced by bHLH and WD40 proteins [16]. Similarly, in *F. hybrida* ‘Red River®’, distinct MBW complexes have been identified, where FhTT8L interacts with FhMYB5 to regulate the general flavonoid pathway and FhPAP1-FhTT8L drives anthocyanin biosynthesis [30, 31]. FhMYBPAs also target PA biosynthetic genes through interactions with FhTT8L (Fig. 4). Notably, the components of the MBW complex engage in mutual transcriptional regulation [15] (Fig. 5), thereby orchestrating the expression of genes associated with various metabolic pathways. Such reciprocal regulation allows for a dynamic response to environmental signals and developmental cues, ensuring that the synthesis of anthocyanins, PAs, and flavonols is finely tuned.

MYB transcription factors can also repress MBW activity by competing with activators for bHLH binding, thus balancing metabolic flux. In petunia, PhMYB27 and PhMYBx repress anthocyanin biosynthesis by inhibiting the formation of MBW-activating complex [53]. Remarkably, the activating complex positively regulates *PhMYB27* and *PhMYBx*, forming a feedback loop that tightly controls anthocyanin production [53]. Similarly, the identification of MYB inhibitors of PA biosynthesis, like VvMYBC2 in grapes and PtMYB182 in poplar, suggests a similar feedback loop in PA biosynthesis regulation among angiosperms [20, 54]. In *F. hybrida*, PA-related MYB activators FhMYBPAs coordinate a network involving FhTTG1 and repressors FhMYB27 and FhMYBx (Fig. 5), underscoring the complexity of PA biosynthesis and regulation. Despite identifying several PA-related MYB activators in *F. hybrida*, the role of tissue-specific regulatory networks remains uncertain considering specific regulatory systems governing the anthocyanin biosynthesis in soybean seeds and flowers [55, 56].

### Pleiotropic and specialized regulation of flavonoid biosynthesis during *Freesia* flower development process

PAs, anthocyanins, and flavonols share significant portions of their synthetic pathways, resulting in complex interactive relationships. The competition among enzymes such as DFR and FLS, LAR and ANS, and ANR and GT, for common substrates, significantly influences the flux of those metabolites. For instance, in grape and raspberry, PA levels decline as fruit matures, while anthocyanin content increases. Correspondingly, genes controlling PA accumulation are highly expressed earlier during fruit development, while those regulating anthocyanin biosynthesis are upregulated after fruit coloring [57, 58]. In *Freesia*, preliminary research suggests a similar relationship between PA and anthocyanin levels and their regulatory genes (Fig. 2). Considering the predominant flavonol and anthocyanin biosynthesis in *Freesia* flowers [31, 32], a finely tuned regulatory network may exist in coordinately governing PA, flavonol, and anthocyanin accumulation during *Freesia* flower development.

Increasing evidence demonstrates versatile transcription factors regulating diverse metabolites [59]. Given that PAs, anthocyanins, and flavonols share much of the biosynthetic pathway, the cooperative regulation of these metabolites, and whether MYBs previously thought to target specific metabolites might also regulate others in particular organs like flowers, remains largely elusive. While MYBs retain the capacity to regulate the early shared-pathway genes, they also demonstrate specific activations on branching genes responsible for specific metabolites (Fig. 6). However, pleiotropic functions are also observed for some MYBs, such as FhMYB5 and FhMYB21 (Fig. 6), and their orthologs in other species, suggesting complex regulatory mechanisms underlying metabolite biosynthesis [27, 32, 60–63].

**Figure 7:**
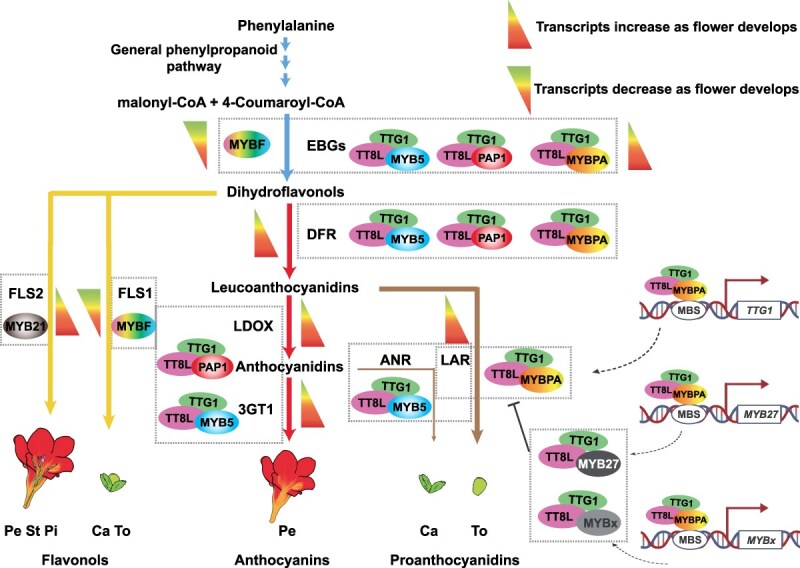
Proposed model of flavonoid biosynthesis regulation in *Freesia* flower. Various MYB transcription factors regulate the flavonoid pathways, either independently or in complexes with bHLH (TT8L) and WD40 (TTG1) proteins. In early flower buds, MYBF alone positively regulates the expression of EBGs, while in mature flowers, the MYB5/PAP1/MYBPA-TT8L-TTG1 complexes regulate these genes. These regulators exhibit distinct roles in the biosynthesis of specific metabolites. Flavonol biosynthesis in the *Freesia* calyx and torus is primarily governed by FLS1, regulated by MYBF, while FLS2, regulated by FhMYB21, controls flavonol biosynthesis in the petals, stamens, and pistils. The MYB5/PAP1/MYBPA-TT8L-TTG1 complexes are also involved in the activation of DFR in anthocyanin and PA biosynthesis. PAP1-TT8L-TTG1 and MYBPA-TT8L-TTG1 differentiate into anthocyanin biosynthesis in petals and PA biosynthesis in the torus, respectively. In comparison, MYB5-TT8L-TTG1 plays versatile roles in both anthocyanin biosynthesis in petals and PA biosynthesis in the calyx. Hierarchical and feedback mechanisms also exist in anthocyanin (Li et al., 2020) and PA biosynthesis. For instance, MYBPA can activate the expression of *TTG1*, *MYB27*, and *MYBx*. TTG1, in turn, promotes the formation of MYBPA-TT8L-TTG1 complex, enhancing PA biosynthesis. Conversely, MYB repressors like MYB27 or MYBx inhibit PA biosynthesis by disrupting the MYBPA-TT8L-TTG1 complex. EBGs, early biosynthetic genes; FLS, flavonol synthase; DFR, dihydroflavonol reductase; LDOX, leucoanthocyanidin dioxygenase; 3GT1, 3-O-glycosyltransferase 1; LAR, anthocyanidin reductase; ANR, anthocyanidin reductase. Ca, calyx; To, torus; Pe, petal; St, stamen; Pi, pistil.

In conclusion, we propose a model for flavonoid biosynthesis regulation in *Freesia* flowers, based on patterns of metabolite accumulation, gene expression profiles, and the distinct regulatory effects of different MYBs on EBGs, LBGs, and flavonoid branch pathway-specific genes (Fig. 7). The identified MBW activator complex, along with MYB repressors and their regulatory loops, highlights the sophisticated mechanisms plants use to precisely control metabolite production. Our study illuminates the complexity of floral flavonoid biosynthesis in *F. hybrida* and sheds light on potential cross-talk among metabolic pathways in plants. Understanding these interactions is crucial for unraveling the broader physiological implications and manipulating potential applications in crop improvement and metabolic engineering.

## Materials and methods

### Plant material and growth conditions

*Freesia hybrida* ‘Red River®’ was cultivated under a controlled 14-h light/10-h dark photoperiod at 15°C. *Arabidopsis thaliana* and *N. tabacum* ‘K326’ plants were grown in a greenhouse illuminated by white fluorescent lamps, maintaining a temperature range of 21°C–22°C during the 16-h day period and 8-h night period.

Young inflorescence segments from *F. hybrida* ‘Red River®’ were aseptically treated and utilized as explants to induce calli, following established protocols demonstrated in modern cultivars [64, 65]. These calli were subsequently utilized for protoplast isolation and transient transfection analysis [65].

The development of *F. hybrida* flowers was categorized into five developmental stages: Stage 1 (S1) featured buds measuring <10 mm in length and lacking pigmentation. Stage 2 comprised buds 10–20 mm long with slight pigmentation, while Stage 3 included buds 20–30 mm long exhibiting pigmentation. Stage 4 encompassed fully pigmented flowers before complete opening, and Stage 5 included fully opened flowers. In *Freesia* flowers, the torus is enclosed by the calyx, with petals, pistil, and stamens attached to the torus. Further dissection of Stage 5 flowers was conducted to isolate calyx, petal, pistil, stamen, and torus components.

For transcriptome analysis, samples from different developmental stages were collected and subjected to analysis by Biomarker Technologies (Beijing, China). Illumina platform-based transcriptome analysis was performed according to the company’s standard procedures. *De novo* transcriptome assembly employed the classical Trinity approach, comprising Inchworm, Chrysalis, and Butterfly, following the published instructions [66].

### Gene cloning and sequence analysis

PA-related MYBs in *F. hybrida* were identified through a homology-based BLAST analysis of the *F. hybrida* transcriptome database in TBtools [67], using an E-value threshold of 1e-5. Protein sequences of *Arabidopsis* AtTT2 (Q9FJA2) and *V. vinifera* VvMYBPA1 (AM259485) served as bait sequences. The identified sequences were further annotated using the NCBI BLAST analysis to predict coding sequences (CDSs). Specific primers (Table S2) were designed based on the predicted CDSs, enabling the cloning of candidate PA-related MYB genes via standard PCR. The cloned sequences were inserted into the p*ESI-Blunt* vector (Hieff Clone Zero TOPO-Blunt Cloning Kit, Yeasen, Shanghai, China) for sequence verification. To assess conservation and divergence among the cloned sequences, multiple sequence alignments were conducted using the Clustal Omega algorithm (https://www.ebi.ac.uk/Tools/msa/clustalo/) with default parameters. Phylogenetic tree was reconstructed in MEGA X using a neighbor-joining algorithm with robustness evaluated via bootstrap resampling analysis (1000 replicates).

### Transient protoplast assay

Four-week-old subcultured *Freesia* calluses, 0.5–1 cm in diameter, and rosette leaves from 4-week-old *Arabidopsis* plants were used for protoplast isolation following established protocols [65, 68]. Briefly, protoplasts were filtered through Miracloth (EMD Millipore Corp., Billerica, MA, USA) and pelleted by centrifugation to remove undigested tissues and enzyme solution. They were then transformed with endotoxin-free plasmids using PEG 3350. After 20–22 h of incubation in the dark, protoplasts were harvested by centrifugation for further analysis.

For subcellular localization or BiFC analysis, protoplasts were visualized using an Olympus FluoView FV1000 confocal microscope (Olympus, Japan). Excitation/emission wavelengths of 488/493–556 nm were used for GFP constructs, and 561/590–628 nm for RFP constructs, respectively. Image processing, including contrast and brightness corrections, was done with ImageJ software (NIH, USA).

For GUS detection, Luciferase Cell Culture Lysis 5 × Reagent (Promega, Madison, WI) was used to lyse protoplasts. 10 μl of protoplast lysate was incubated with 100 μl of MUG (4-methylumbelliferyl glucuronide; Gold BioTechnology, Inc.) substrate mix containing 1 mM MUG and 2 mM MgCl_2_ in 10 mM Tris–HCl (pH 8.0) for 1 h. GUS activities were measured using an Agilent BioTek Synergy HTX Multi-Mode Microplate Reader (USA) after adding 100 μl of 0.6 M Na_2_CO_3_ to stop the reaction.

For the dual-luciferase reporter assay, luminescence detection was performed using the Dualucif® Firefly & Renilla Assay Kit (US Everbright® Inc., Suzhou, China) following the manufacturer’s instructions. Luminescence measurements were recorded using a GENios Pro TECAN instrument (Guangzhou, China).

For RT-qPCR assay, RNA extraction from protoplasts was performed using the OminiPlant RNA Kit containing DNase I (CWBIO, Beijing, China). Two micrograms of RNA was reverse-transcribed into cDNA using All-In-One 5× RT MasterMix (ABM, Richmond, Canada) in a final volume of 20 μl, which was then diluted to 100 μl for further gene cloning and expression analysis. RT-qPCR analysis was carried out with TB Green® Premix Ex Taq™ II (Tli RNaseH Plus, Takara Bio USA, Inc.) in a total volume of 10 μl. *Fhactin* and *Fhubiquitin* genes were used as internal controls, and relative expression levels were calculated with the 2^-ΔΔC^_t_ method [69]. Additional parameters can be referenced from previously published papers [32].

### Yeast two-hybrid assay

Bait and prey vectors were cotransformed into the yeast strain AH109 utilizing the lithium acetate/single-stranded DNA/PEG400 method. Positive transformants were confirmed on selective plates containing SD/−Trp-Leu. Further validation of the interaction was performed on plates supplemented with SD/−Trp-Leu-His-Ade.

### Genetic transformation

For agrobacterium infection, leaves from 6- to 8-week-old *N. tabacum* were used, following previously established methods [70]. In summary, 4 days postinfiltration, tobacco leaves were excised and cultured on medium to generate transgenic seedlings. These seedlings were then transferred to soil and maintained in a greenhouse until transgenic flowers fully bloomed. Harvested flowers from the first day of full bloom were used for PA detection or divided into five distinct tissues: toruses, calyxes, petals, stamens, and pistils for staining analysis or RT-qPCR analysis. For transient gene expression in *F. hybrida*, agrobacterium containing different vectors was injected into buds at Stage 2 [31]. After 4 or 5 days of infiltration, the petals were subjected to PA and gene expression analysis.

### PA analysis

To determine PA contents, the methodology outlined in a previous study [30], was employed. Briefly, samples were extracted using an aqueous acetone solution. Following centrifugation, the supernatant was combined with diethyl ether for phase separation. The lower phase was then mixed with DMACA reagent and quantified as A640 g^−1^ fresh weight (FW).

Histochemical staining of PAs in tobacco flowers was conducted following established procedures [31]. Tissues were stained with a 0.3% (w/v) DMACA solution dissolved in ethanol: 6 M HCl (1:1) for 30 min, followed by washing with 70% ethanol.

### Electrophoretic mobility shift assay

For EMSA, pGEX-4 T-2-FhMYBPA2 was transformed into *Escherichia coli* BL21. The resulting recombinant FhMYBPA2 fused with GST was induced using isopropyl-β-d-thiogalactopyranoside (IPTG) and purified with immobilized glutathione beads (Sangon Biotech Co. Ltd, Shanghai, China). Subsequent procedures were performed using the EMSA kit (Beyotime, Shanghai, China) according to the provided instructions. Further details can be found in our earlier publications [27, 32].

### Vector construction

All vector constructions utilized the pEASY®-Basic Seamless Cloning and Assembly Kit (TransGen Biotech, Beijing, China) following the manufacturer’s instructions.

For subcellular localization studies, the respective CDS was subcloned into *Nde* I- and *Cla* I-digested *35S: FhMYB27-GFP* vector [34], yielding *35S: FhMYBPAs-GFP.*

BiFC assay vectors incorporated seamless cloning of the CDSs of *FhMYBPAs* into previously utilized *35S: GFPC-FhTTG1* [31].

Vectors for evaluating transactivation capacities of FhMYBPAs or interactions with FhTT8L in protoplasts involved subcloning of the respective CDS into the *Nde* I- and *Afl* II-digested *35S: GD-FhMYBF* or *35S: HA-FhMYBF* [32], creating constructs like *35S: GD-FhMYBPAs* or *35S: HA-FhMYBPAs.*

For vectors used in plant transformation, the CDSs of *FhMYBPAs* were seamlessly cloned into *Bam*HI- and *Sac*I-digested *pBI121* binary vector*.*

For reporter plasmids such as *TTG1-F_pro_:GUS, MYB5-F_pro_:GUS*, the 869-bp and 1088-bp regions upstream of the initiation codon ATGs were regarded as promoters and subcloned into *Pst* I- and *Sac* I-digested *GmF3′H(P3)_pro_:GUS*, respectively [55].

Vectors designed for luciferase assays incorporated the 1744-bp and 1910-bp regions upstream of the inition codon ATGs of *FhLAR* and *FhANR* to promote the *luciferase* gene in the backbone of *pGreenII 0800-LUC*.

In yeast hybrid experiments, the sequences of *FhMYBPAs*, *FhMYBF1*, *FhMYB5*, *FhPAP1*, and *FhMYB21L2* were subcloned into *Nde*I- and *Xma*I-digested *pGADT7,* respectively*,* while *FhTT8L* was cloned into *Nde* I- and *Xma* I-digested *pGBKT7.*

For vectors utilized in EMSA, the CDS of FhMYBPA2 was subcloned into the previously used *pGEX-4 T-2-GmMYBA2* vector with *Bam*H I and *Xho* I, resulting in *pGEX-4 T-2-FhMYBPA2*.

Please refer to our earlier published papers for additional details on other plasmids used in these experiments [31–34].

## Supplementary Material

Web_Material_uhae352

## Data Availability

All data supporting the findings of this study are available within the paper and within its Supplemental data published online.
