# Supplementary material for: Molecular insights into TT2-type MYB regulators illuminate the complexity of floral flavonoids biosynthesis in Freesia hybrida
Source: Hortic Res. 2024 Dec 12;12(3):uhae352. doi: 10.1093/hr/uhae352 (PMC11879501; doi:10.1093/hr/uhae352)
Supplement: Web_Material_uhae352 [file web_material_uhae352.docx]

**Molecular Insights into TT2-Type MYB Regulators** **Illuminate** **the Complexity of Floral Flavonoids Biosynthesis in *Freesia hybrida***

Xiaotong Shan^1^, Deyu Zhuang^1^, Ruifang Gao^1, 2^, Meng Qiu^1^, Liudi Zhou^1^, Jia Zhang^1^, Yanan Wang^1^, Qi Zhang^1^, Niu Zhai^3^, Guoyun Xu^3^, Li Wang^1^, Yueqing Li^1,*^, Xiang Gao^1,*^

^1^Key Laboratory of Molecular Epigenetics of MOE and Institute of Genetics & Cytology, Northeast Normal University, Changchun 130024, China

^2^College of Plant Science, Jilin University, Changchun 130062, Jilin, China

^3^China Tobacco Gene Research Center, Zhengzhou Tobacco Research Institute of CNTC, Zhengzhou, 450001, China

*Corresponding authors.

**Email address for each author:**

**Xiaotong Shan**: [danxt705@nenu.edu.cn;](mailto:danxt705@nenu.edu.cn;) **Deyu Zhuang**: [zhuangdy064@nenu.edu.cn;](mailto:zhuangdy064@nenu.edu.cn;) **Ruifang Gao**: [gaorf715@jlu.edu.cn;](mailto:gaorf715@jlu.edu.cn;) **Meng Qiu**: [qium050@nenu.edu.cn;](mailto:qium050@nenu.edu.cn;) **Liudi Zhou**: [zhould243@nenu.edu.cn;](mailto:zhould243@nenu.edu.cn;) **Jia Zhang**: [zhangj656@nenu.edu.cn;](mailto:zhangj656@nenu.edu.cn;) **Yanan Wang**: [wangyn517@nenu.edu.cn;](mailto:wangyn517@nenu.edu.cn;) **Qi Zhang**: [qizhang@nenu.edu.cn;](mailto:qizhang@nenu.edu.cn;) **Niu Zhai**: [zhain@ztri.com.cn;](mailto:zhainiu2005@163.com;) **Guoyun Xu**: [xugy@ztri.com.cn;](mailto:xgyun1018@163.com;) **Li Wang**: [wangli@nenu.edu.cn;](mailto:wangli@nenu.edu.cn;) **Yueqing Li**: [liyq339@nenu.edu.cn;](mailto:liyq339@nenu.edu.cn;) **Xiang Gao**: [gaox891@nenu.edu.cn;](mailto:gaox891@nenu.edu.cn;) [gaoxiang424@163.com](mailto:gaoxiang424@163.com;)

**Running Title**

Complexity of floral flavonoid biosynthesis in *Freesia*

**Table S1. Information of *FhMYBPAs* genes**

| **Candidate transcripts** | **Protein sequence length** | **Top BLAST match** | **Homology (%)** | **GenBank number of the homology genes used in Blast** |
| --- | --- | --- | --- | --- |
| *FhMYBPA1* | 247 residues | UTU02183.1  MYBPA2  *Musa acuminata* AAA Group | 70 | Q9FJA2/  AM259485 |
| *FhMYBPA2* | 278 residues | RVW44110.1 transcription factor TT2  *Vitis vinifera* | 58 | Q9FJA2/  AM259485 |
| *FhMYBPA3* | 254 residues | UTU02182.1 MYBPA1  *Musa acuminata* AAA Group | 67 | Q9FJA2/  AM259485 |
| *FhMYBPA4* | 278 residues | UTU02182.1 MYBPA1  *Musa acuminata* AAA Group | 62 | Q9FJA2/  AM259485 |

**Table S2: Primers used in the study**

|  | | Forward (5'-3') | Reverse (5'-3') |
| --- | --- | --- | --- |
| Full length cDNA sequence cloning |  |  |  |
|  | *FhMYBPA1* | ATGGGAAGAGAGCCATGTTGT | TTAAAACCATTGGTCCTC |
|  | *FhMYBPA2* | ATGGTGAAGAAGGCTTG | CTAGAGGAAACCACCCTGA |
|  | *FhMYBPA3* | ATGGGCAGGAGGCCTTGTT | TTAGACACAAGAAGGCCTTCTCA |
|  | *FhMYBPA4* | ATGGGGAGGAGGCCTTGTT | TTACGACACAGATGAAGGCCCT |
| Protein purification |  |  |  |
|  | *PGEX-4T-2- FhMYBPA2* | TGGTTCCGCGTGGATCCATGGTGAAGAAGGCTTG | CGATGCGGCCGCTCGAGCTAGAGGAAACCACCCTGA |
| Plant transformation |  |  |  |
|  | *pBI121-FhMYBPA1* | GGGACTCTAGAGGATCCATGGGAAGAGAGCCATGTTGT | GATCGGGGAAATTCGAGCTCTTAAAACCATTGGTCCTC |
|  | *pBI121-FhMYBPA2* | GGGACTCTAGAGGATCCATGGTGAAGAAGGCTTG | GATCGGGGAAATTCGAGCTCCTAGAGGAAACCACCCTGA |
|  | *pBI121-FhMYBPA3* | GGGACTCTAGAGGATCCATGGGCAGGAGGCCTTGTT | GATCGGGGAAATTCGAGCTCTTAGACACAAGAAGGCCTTCTCA |
|  | *pBI121-FhMYBPA4* | GGGACTCTAGAGGATCCATGGGGAGGAGGCCTTGTT | GATCGGGGAAATTCGAGCTCTTACGACACAGATGAAGGCCCT |
| Transient Expression Assay |  |  |  |
|  | *GD-FhMYBPA1* | CTGTATCGCCGCATATGATGGGAAGAGAGCCATGTTGT | AGGATTCAATCTTAAGTTAAAACCATTGGTCCTC |
|  | *GD-FhMYBPA2* | CTGTATCGCCGCATATGATGGTGAAGAAGGCTTG | AGGATTCAATCTTAAGCTAGAGGAAACCACCCTGA |
|  | *GD-FhMYBPA3* | CTGTATCGCCGCATATGATGGGCAGGAGGCCTTGTT | AGGATTCAATCTTAAGTTAGACACAAGAAGGCCTTCTCA |
|  | *GD-FhMYBPA4* | CTGTATCGCCGCATATGATGGGGAGGAGGCCTTGTT | AGGATTCAATCTTAAGTTACGACACAGATGAAGGCCCT |
|  | *HA-FhMYBPA1* | CTGATTACGCTCATATGATGGGAAGAGAGCCATGTTGT | AGGATTCAATCTTAAGTTAAAACCATTGGTCCTC |
|  | *HA-FhMYBPA2* | CTGATTACGCTCATATGATGGTGAAGAAGGCTTG | AGGATTCAATCTTAAGCTAGAGGAAACCACCCTGA |
|  | *HA-FhMYBPA3* | CTGATTACGCTCATATGATGGGCAGGAGGCCTTGTT | AGGATTCAATCTTAAGTTAGACACAAGAAGGCCTTCTCA |
|  | *HA-FhMYBPA4* | CTGATTACGCTCATATGATGGGGAGGAGGCCTTGTT | AGGATTCAATCTTAAGTTACGACACAGATGAAGGCCCT |
|  | *HA-AtTT2* | CTGATTACGCTCATATGATGGGAAAGAGAGCAACTACTA | AGGATTCAATCTTAAGTCAACAAGTGAAGTCTCGGAG |
|  | *HA-AtTTG1* | CTGATTACGCTCATATGATGGATAATTCAGCTCCAGATTCG | AGGATTCAATCTTAAGTCAAACTCTAAGGAGCTGCATTTTG |
|  | *FhMYBPA1-GFP* | CTGATTACGCTCATATGATGGGAAGAGAGCCATGTTGT | TGCTCACCATATCGATAAACCATTGGTCCTCCGAATC |
|  | *FhMYBPA2-GFP* | CTGATTACGCTCATATGATGGTGAAGAAGGCTTG | TGCTCACCATATCGATGAGGAAACCACCCTGACTC |
|  | *FhMYBPA3-GFP* | CTGATTACGCTCATATGATGGGCAGGAGGCCTTGTT | TGCTCACCATATCGATGACACAAGAAGGCCTTCTCATAT |
|  | *FhMYBPA4-GFP* | CTGATTACGCTCATATGATGGGGAGGAGGCCTTGTT | TGCTCACCATATCGATCGACACAGATGAAGGCCCT |
|  | *GFP(C)- FhMYBPA1* | AGCTGTACAAGCATATGATGGGAAGAGAGCCATGTTGT | AGGATTCAATCTTAAGTTAAAACCATTGGTCCTC |
|  | *GFP(C)- FhMYBPA2* | AGCTGTACAAGCATATGATGGTGAAGAAGGCTTG | AGGATTCAATCTTAAGCTAGAGGAAACCACCCTGA |
|  | *GFP(C)- FhMYBPA3* | AGCTGTACAAGCATATGATGGGCAGGAGGCCTTGTT | AGGATTCAATCTTAAGTTAGACACAAGAAGGCCTTCTCA |
|  | *GFP(C)- FhMYBPA4* | AGCTGTACAAGCATATGATGGGGAGGAGGCCTTGTT | AGGATTCAATCTTAAGTTACGACACAGATGAAGGCCCT |
|  | *MYB5-F_pro_:GUS* | CCAAGCTTGCATGCCTGCAGCGTCTAGAATGTTCCTTAAACC | GCTAAGCTTACCATGAGCTCCGATCCCGATGACTGATTATT |
|  | *TTG1-F_pro_:GUS* | CCAAGCTTGCATGCCTGCAGGAAAAAAAAAAGGAGATGA | GCTAAGCTTACCATGAGCTCGGGGGAGTTGTCCATTTT |
|  | *TTG1-F_pro(-690)_:GUS* | CCAAGCTTGCATGCCTGCAGAATAACTGATCAGAGA | GCTAAGCTTACCATGAGCTCGGGGGAGTTGTCCATTTT |
|  | *FLS1-F_pro(-411)_:GUS* | CCAAGCTTGCATGCCTGCAGAAGAACAAATTCTCTA | GCTAAGCTTACCATGAGCTCGGGGGAGTTGTCCATTTT |
|  | *FLS1-F_pro(-161)_:GUS* | CCAAGCTTGCATGCCTGCAGACTCGATTGCGCGCG | GCTAAGCTTACCATGAGCTCGGGGGAGTTGTCCATTTT |
| Dual-luciferase assay |  |  |  |
|  | *LAR-Fpro:LUC* | TCGACGGTATCGATAAGCTTCCAGCTTGAACCCGCTAAAGT | GCTCTAGAACTAGTGGATCCTAACGGGCCGAGTTCATGAGTA |
|  | *ANR-Fpro:LUC* | TCGACGGTATCGATAAGCTTTGGGCCTGATCCTAGAATAAG | GCTCTAGAACTAGTGGATCCTCTCTTCCTCTGCATACAACTCA |
| Y2H assay |  |  |  |
|  | *AD-FhMYBPA1* | CAGATTACGCTCATATGATGGGAAGAGAGCCATGTTGT | TCGATGCCCACCCGGGTTAAAACCATTGGTCCTC |
|  | *AD-FhMYBPA2* | CAGATTACGCTCATATGATGGTGAAGAAGGCTTG | TCGATGCCCACCCGGGCTAGAGGAAACCACCCTGA |
|  | *AD-FhMYBPA3* | CAGATTACGCTCATATGATGGGCAGGAGGCCTTGTT | TCGATGCCCACCCGGGTTAGACACAAGAAGGCCTTCTCA |
|  | *AD-FhMYBPA4* | CAGATTACGCTCATATGATGGGGAGGAGGCCTTGTT | TCGATGCCCACCCGGGTTACGACACAGATGAAGGCCCT |
|  | *BD-FhTT8L* | AGGAGGACCTGCATATGATGGCGTCGCAGCAGTCGAG | TCGACGGATCCCCGGGCTAGTATTGGGAGAAGATATG |
| qRT-PCR |  |  |  |
|  | *FhCHS1* | GAGCTACTAGGCATGTTCTCAGCG | CCTTCTCCGGTCGTACCGTTCT |
|  | *FhCHI2* | CCTCCATCCTCTTCACTCATTC | CACAGTCAGCATTGCGTTATC |
|  | *FhF3H* | ATACCCGTGATTTCGTTGG | CGAACCTCAACTTGTCCTCC |
|  | *FhF3’H* | GCACAAAGCCTCACCAGAC | TAGGGGGCGAAAACCAAGT |
|  | *FhF3’5’H* | TCTTCATCTCTGCCATTGTCT | AGATTGCCGAGGATAGGAT |
|  | *FhDFR1/2* | TGGAGGAGATGTTCGATGAGGC | TAACCATAGCTACTTCCTCTCCGAC |
|  | *FhDFR3* | TGGCAGAGTACGACGAGAAC | CCCACAACTAAGGTAGGAATGAT |
|  | *FhLDOX1* | AAGGTGCGTATTTCGTGGG | TTCTGCTGGATGTGCTGTG |
|  | *FhLDOX2* | GAGGTCAAGAAGTCGGAGGA | CCCGTGGTTCACAATATGC |
|  | *Fh3GT1* | ACTGCGGATGGAACTCTGTC | TTTCCAAACATGCGACACG |
|  | *FhLAR* | CCCATGACATCTTCATAAACGG | TCTCAGGGTAGAGGGAACTCAC |
|  | *FhANR* | ACGAATCATCTTGCGTTTGTGC | GTTCCTGGCTGTGAGCAAATC |
|  | *18S rRNA* | TCCTGATACGGGGAGGTAGTGACA | ACTTGCCCTCCAATGGATCCTCG |
|  | *AtCHS* | GGCAAAGAAGCGGCAGTGAAG | CGGAAGGACGGAGACCAAGAAG |
|  | *AtCHI* | CTCTCTTACGGTTGCGTTTTCG | CACCGTTCTTCCCGATGATAGA |
|  | *AtF3H* | GACCAAGTCGGTGGATTACAAGC | TCCTTCAACAGGCTGAACCG |
|  | *AtF3’H* | TTCCTTACCTTCAGGCGGTTATC | CGAGAGTGGTGTTGGTGGATG |
|  | *AtDFR* | CTTTGTTCGTGCCACCGTTCG | TCCTTCCTCAGATAAATCAGCCTTCC |
|  | *AtANS* | GTTTGCAGCTTTTCTACGAGG | TGAGCAAAAGTCCGTGGAGG |
|  | *AtBAN* | AACAACTAAATCTCTATCTCTGTA | GAATGAGACCAAAGACTCATATAC |
|  | *AtACTIN* | GCTGAGAGATTCAGATGCCCA | GTGGATTCCAGCAGCTTCCAT |
|  | *NtDFR* | TAAGAAGATGACAGGATGGATG | TGGCGGTATGATGCTAATG |
|  | *NtLAR* | TCAAGGTCCTTTACGCCATC | ACGAACCTGCTTCTCTTTGG |
|  | *NtANR* | TGTTCCCACTTGGGATGATA | TGCACCTATACTCTGTTAGTGGC |
|  | *NtTub1* | TCCGTGGTGATGTTGTG | TGGTGGCTGATAGTTGATAC |
| EMSA |  |  |  |
|  | *FhDFR3* | GTAATTAGCATATATGGATAGAATTGCATTTAATT | AATTAAATGCAATTCTATCCATATATGCTAATTAC |
|  | *mFhDFR3* | GTAATTAGCATATATGAACAGAATTGCATTTAATT | AATTAAATGCAATTCTGTTCATATATGCTAATTAC |
|  | *FhTTG1* | TCAAATCCCAAATGCAACAGAATAACTGATCAGA | TCTGATCAGTTATTCTGTTGCATTTGGGATTTGA |
|  | *mFhTTG1* | TCAAATCCCAAATGCAGTAGAATAACTGATCAGA | TCTGATCAGTTATTCTACTGCATTTGGGATTTGA |

Underlined sequences indicate the cutting sites of restriction enzymes.

**
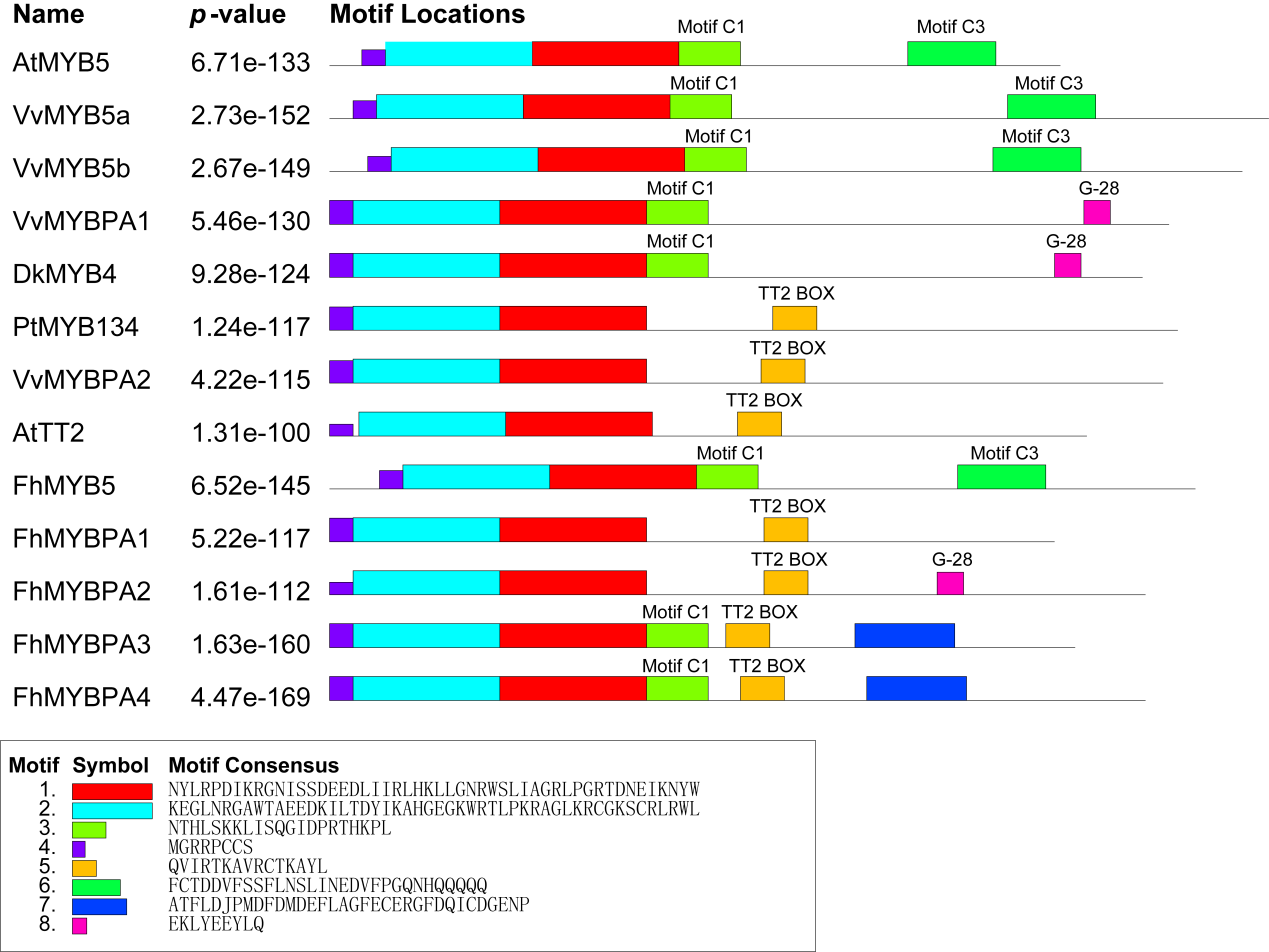
**

**Figure S1. Motif analysis of FhMYBPAs and subgroup 5 of R2R3-MYB members.**

The motif analysis were performed by the online software MEME. TT2-type MYBs contain a TT2-box ([R/P]xKAxRC). MYB5-type MYBs contain motif C1 (Lx2QG[I/T]DPxTHK) and motif C3 ([N/D]D[V/K]F[S/T]SFL[N/D]SLI[N/K]). MYBPA-type MYBs contain motif C1 and motif G-28 (L[E/DKLYEEYL[Q/E][L/V]L]).


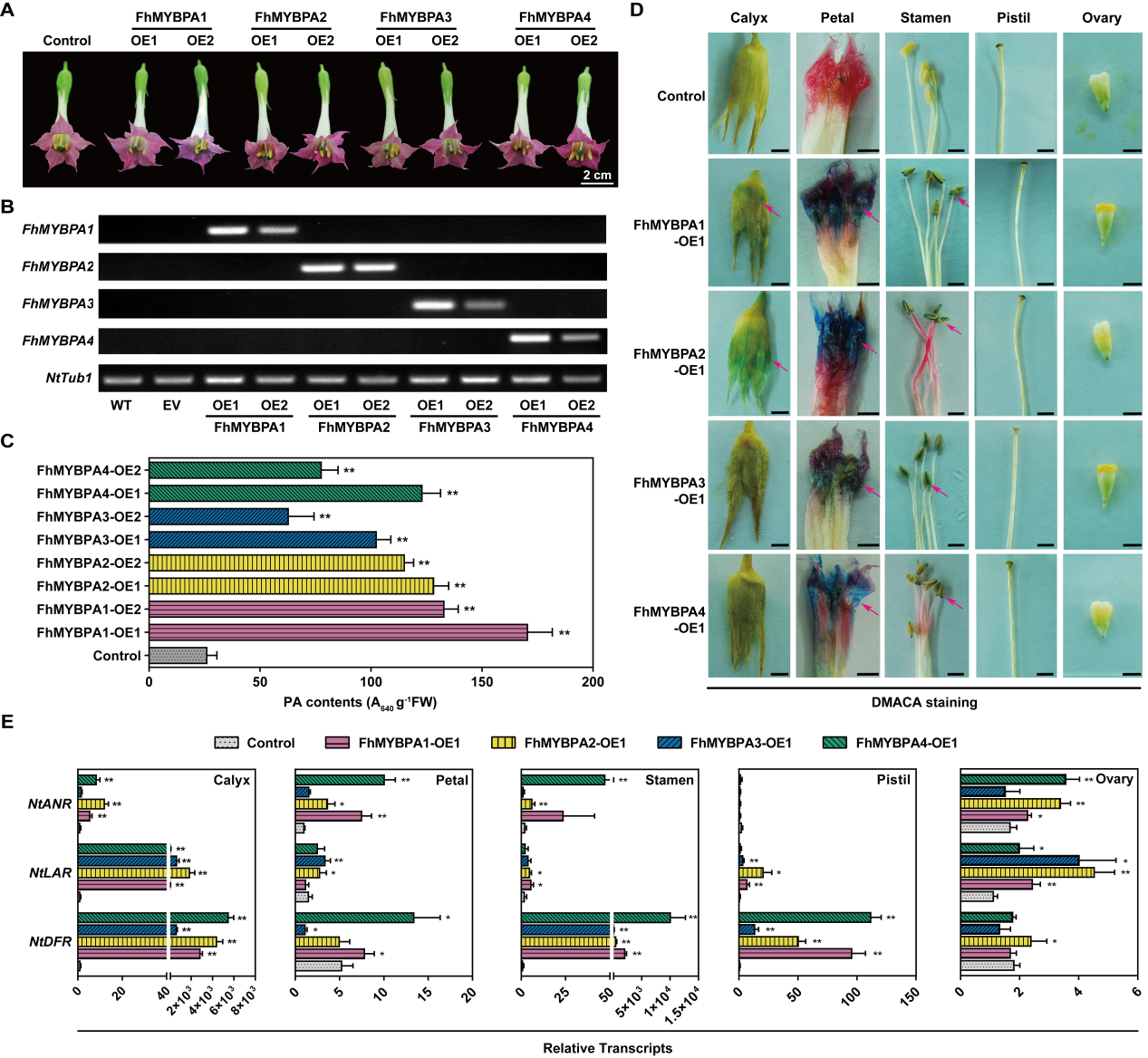


**Figure S2. FhMYBPAs regulate PAs accumulation in transgenic tobacco flowers.**

A and B. Phenotypes and *FhMYBPAs* expression analysis of transgenic tobacco flowers. WT, wild type plant; Control and EV, tobacco expressing empty vector; OE1/2, two transgenic lines over-expressing *FhMYBPAs*.

1. PA contents in transgenic tobacco flowers.
2. DMACA staining showing PA accumulation in various floral tissues isolated from transgenic flowers.
3. Transcript levels of *NtANR*, *NtLAR* and *NtDFR* in tobacco floral tissues overexpressing *FhMYBPAs.*

Data points represent mean±SD of three biological replicates (individual flowers). Student’s *t*-test was used to analyze the significant difference (*, p < 0.05; **, p < 0.01).


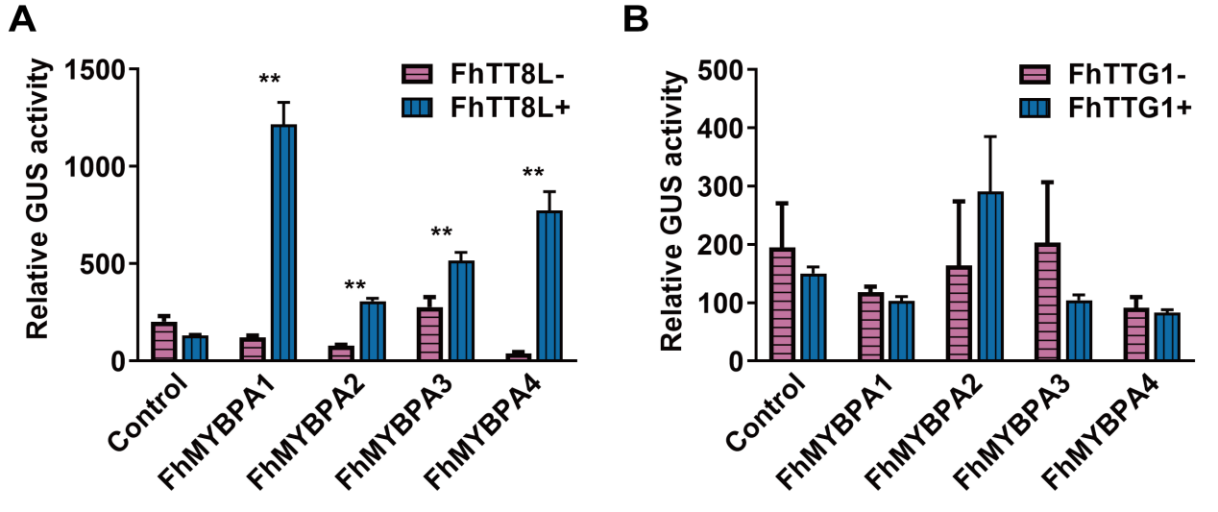


**Figure S3. The interactions between FhMYBPAs and** **flavonoid-related bHLH or WD40 proteins detected by Gal4-based transient protoplast assays.**

1. Interaction between FhMYBPAs and FhTT8L. B. Interaction between FhMYBPAs and FhTTG1. FhMYBPAs were co-expressed with reporter construct GAL4-GUS in the absence or presence of GD-tagged FhTT8L or FhTTG1 proteins. The constructs were co-transfected into Arabidopsis protoplasts and incubated for 20-22 hours before being subjected to GUS detection. Data represent mean ± SD of three replicates. Statistical analysis was performed by Student’s *t*-test (*, P < 0.05; **p < 0.01).


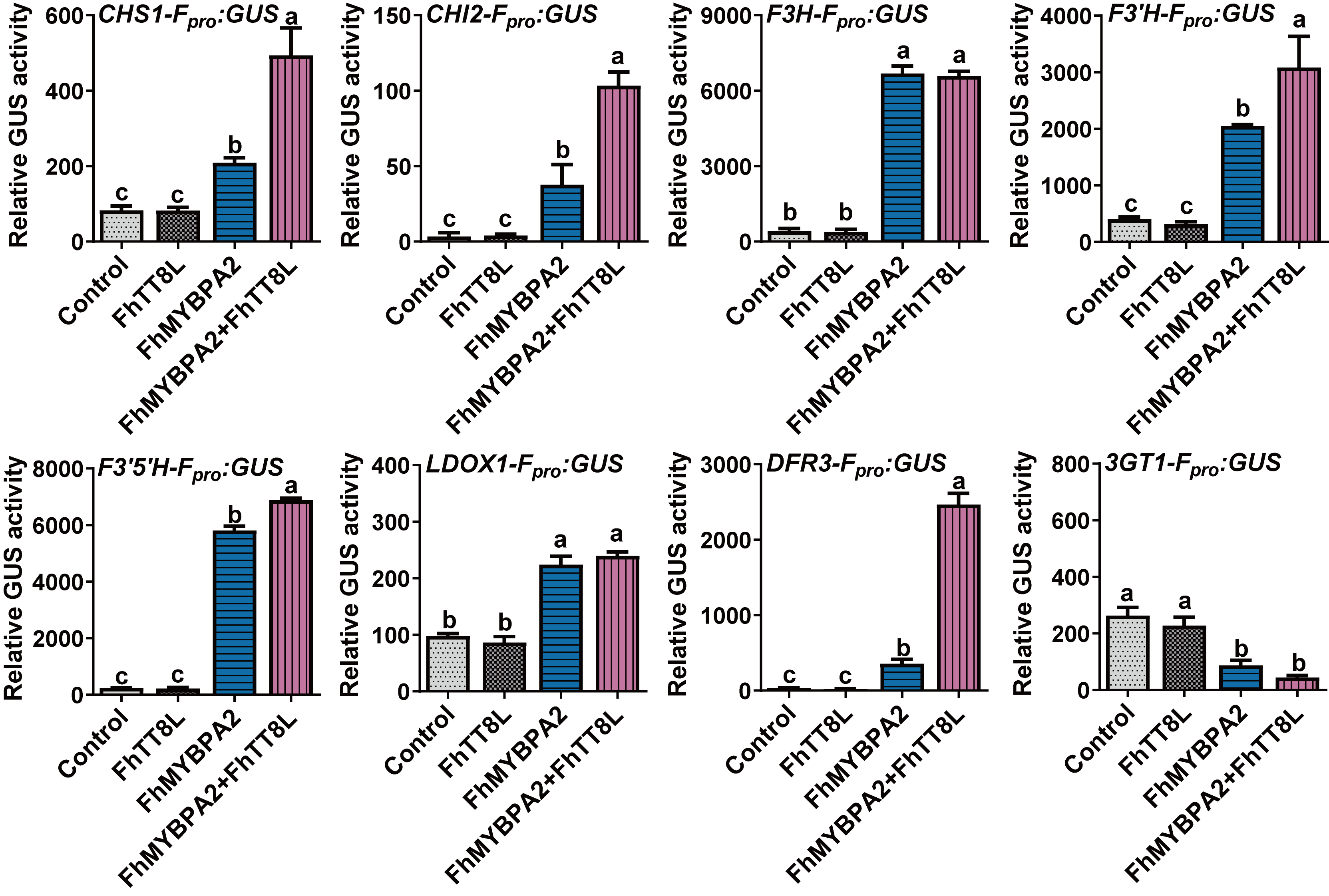


**Figure S4. Activation effects of** **FhMYBPA2 and FhTT8L on promoters of** ***Freesia* flavonoid biosynthetic genes.**

*GUS* was promoted by respective promoter of the *Freesia* flavonoid biosynthetic genes as the reporter. *FhMYBPA2* was transfected alone or co-transfected with *FhTT8L* as effectors. Rosette leaves from 4-week-old Arabidopsis plants were used for protoplast isolation. Each column represents the mean value of three replicates with error bars indicating SD. One-way ANOVA was carried out to compare statistical differences (Ducan, P < 0.05).


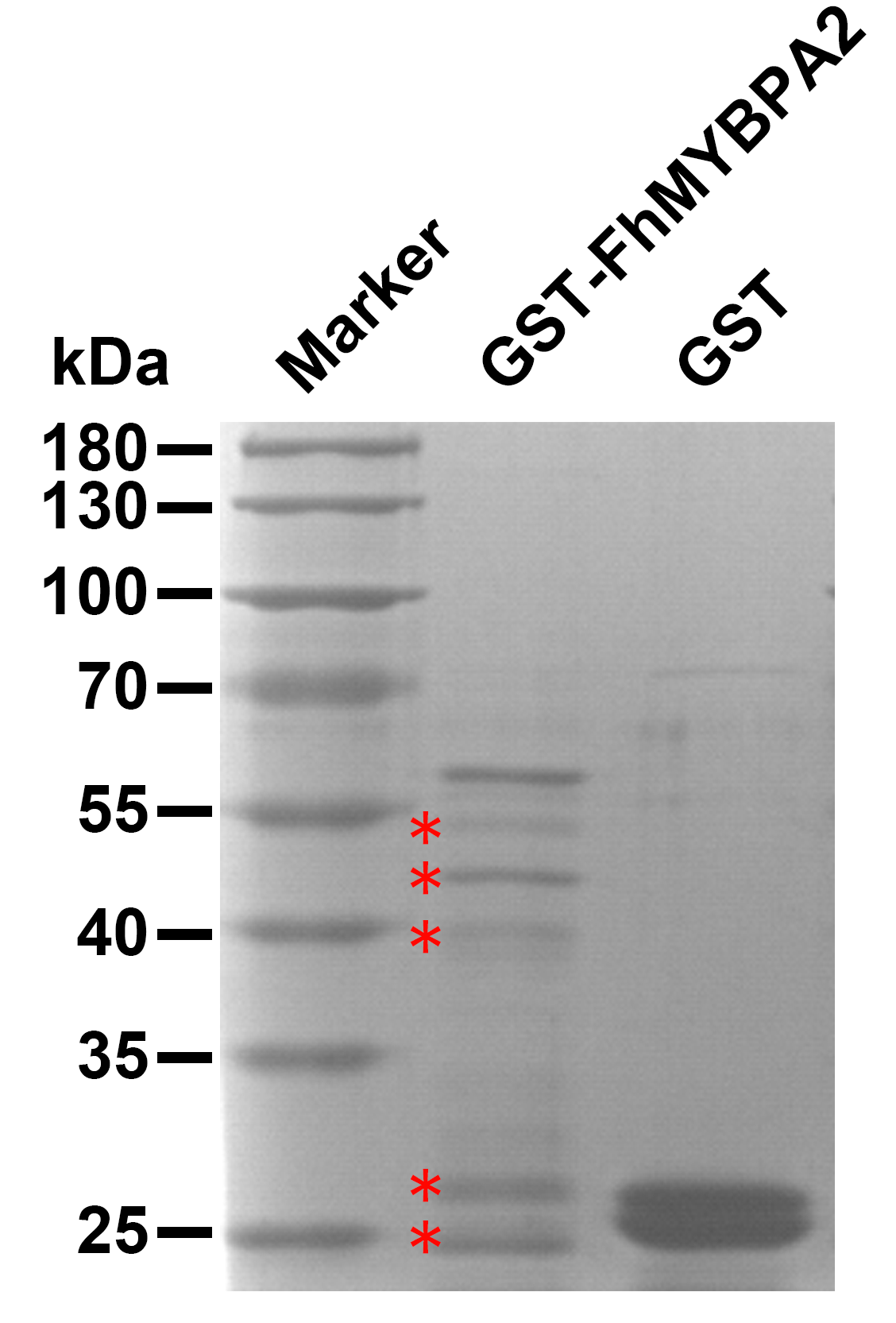


**Figure S5. SDS-PAGE analysis of FhMYBPA2 protein purified from *E. coli* BL21.**

GST and GST-FhMYBPA2 proteins were induced by 0.75 mM IPTG, purified by glutathione-agarose beads and seperated by 10% polyacrylamide gel. Red asterisks indicate broken bands.


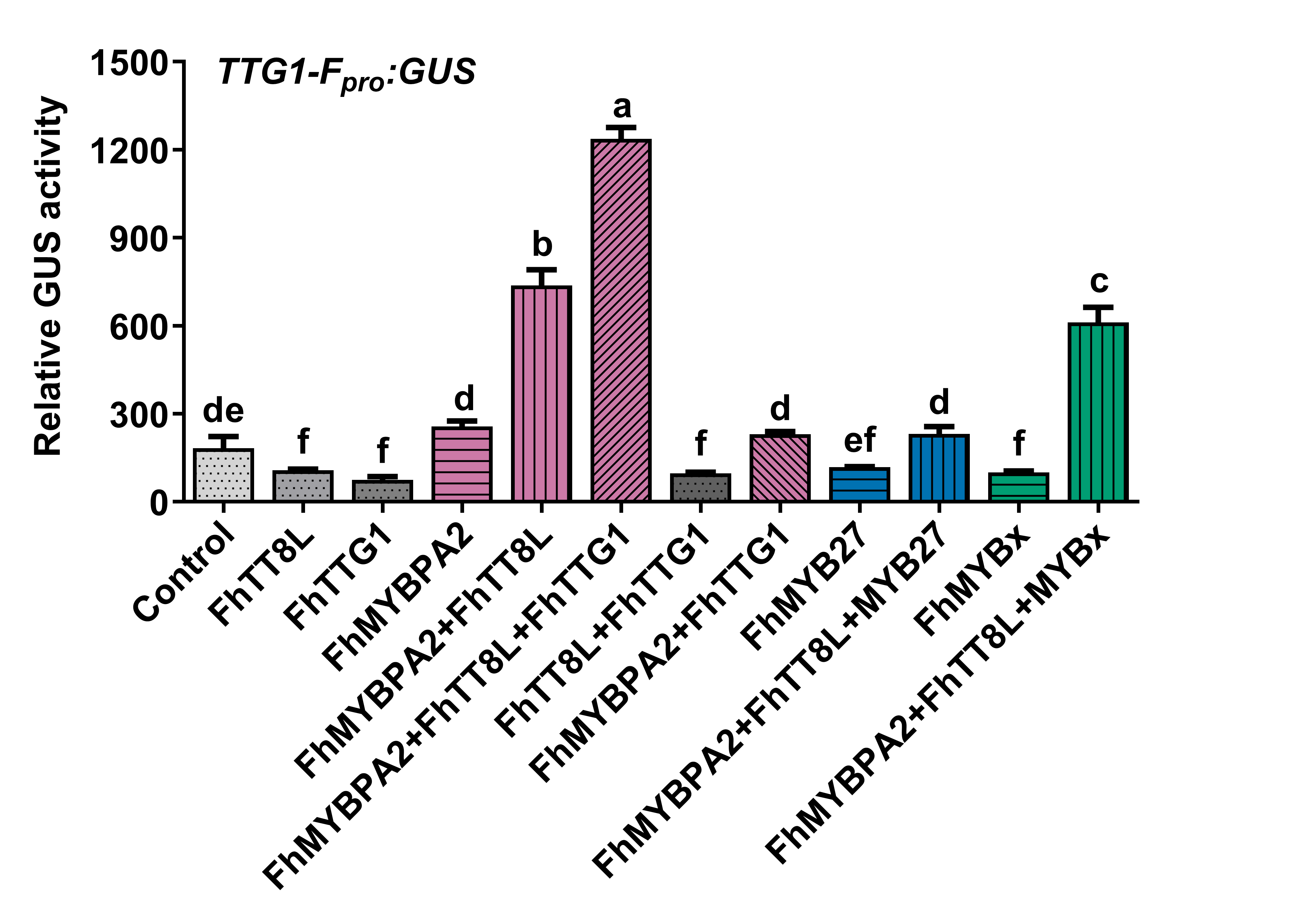


**Figure S6. Transient activation assays of different regulators on *FhTTG1* promoter**.

The reporter construct *TTG1-F_pro_:GUS* contained a *GUS* reporter gene driven by *FhTTG1* promoter. *TTG1-F_pro_:GUS* was cotransfected into Arabidopsis protoplasts together with the effector constructs diagrammed under the pillars. Data represent the mean±SD of three replicates. One-way ANOVA was carried out to compare statistical differences (Ducan, P < 0.05).

**
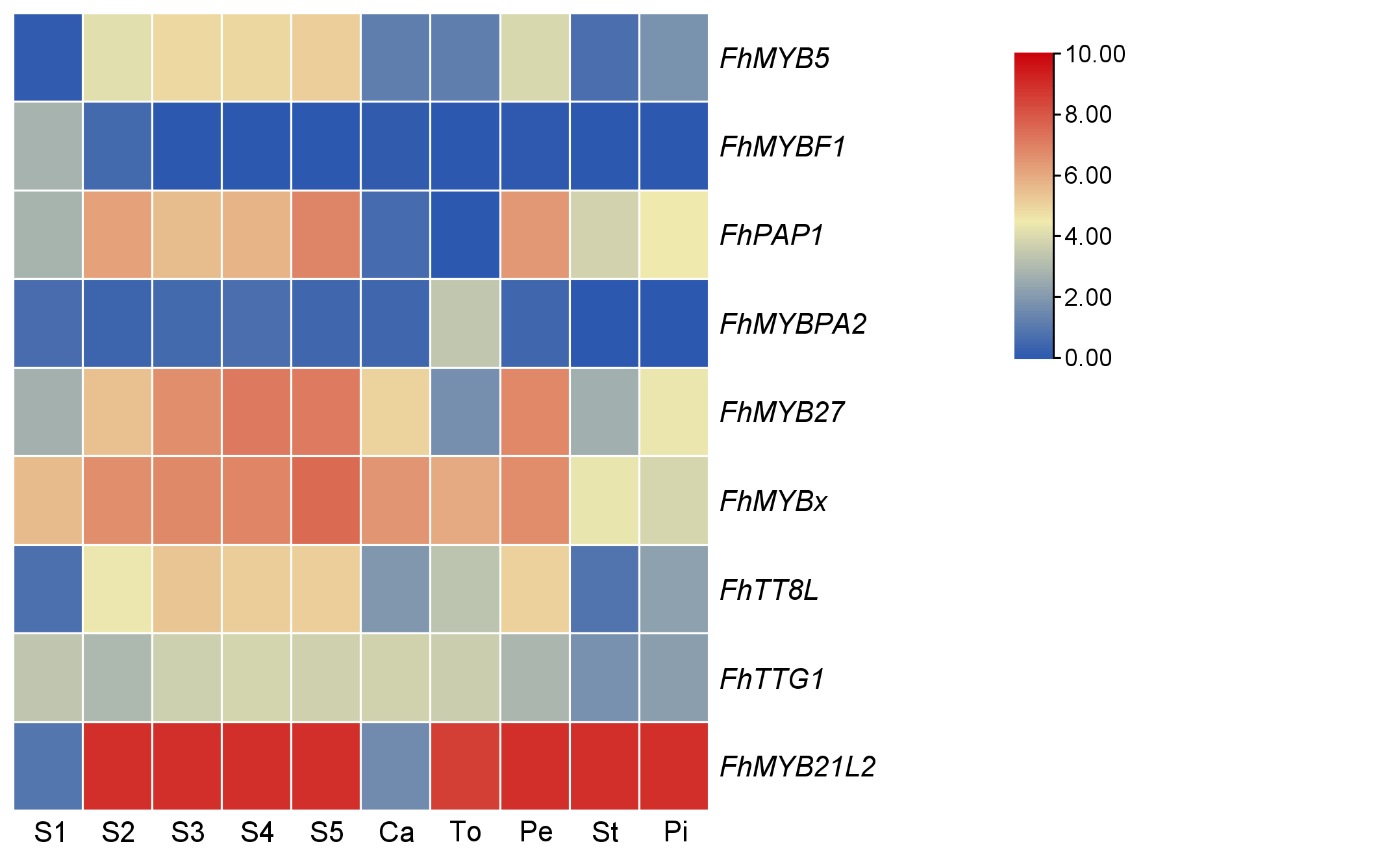
**

**Figure S7. Expression patterns of flavonoid-related MYBs at different flower developmental stages and tissues in *F. hybrida* ‘Red River^®^’.**

FPKM values from RNA-seq were normalized by log_2_(FPKM+1). Red and blue boxes indicated high and low expression levels correspondingly.
